# New Histamine-Related Five-Membered N-Heterocycle Derivatives as Carbonic Anhydrase I Activators

**DOI:** 10.3390/molecules27020545

**Published:** 2022-01-15

**Authors:** Niccolò Chiaramonte, Alessio Gabellini, Andrea Angeli, Gianluca Bartolucci, Laura Braconi, Silvia Dei, Elisabetta Teodori, Claudiu T. Supuran, Maria Novella Romanelli

**Affiliations:** Section of Pharmaceutical and Nutraceutical Sciences, Department of Neuroscience, Psychology, Drug Research and Child Health, University of Florence, Via Ugo Schiff 6, 50019 Florence, Italy; niccolo.chiaramonte@gmail.com (N.C.); alessio.gabellini@unifi.it (A.G.); andrea.angeli@unifi.it (A.A.); gianluca.bartolucci@unifi.it (G.B.); laura.braconi@unifi.it (L.B.); silvia.dei@unifi.it (S.D.); elisabetta.teodori@unifi.it (E.T.)

**Keywords:** carbonic anhydrase activators, heterocycles, histamine-related compounds

## Abstract

A series of histamine (HST)-related compounds were synthesized and tested for their activating properties on five physiologically relevant human Carbonic Anhydrase (hCA) isoforms (I, II, Va, VII and XIII). The imidazole ring of HST was replaced with different 5-membered heterocycles and the length of the aliphatic chain was varied. For the most interesting compounds some modifications on the terminal amino group were also performed. The most sensitive isoform to activation was hCA I (K_A_ values in the low micromolar range), but surprisingly none of the new compounds displayed activity on hCA II. Some derivatives (**1**, **3a** and **22**) displayed an interesting selectivity for activating hCA I over hCA II, Va, VII and XIII.

## 1. Introduction

Carbonic Anhydrases (CAs) are metalloenzymes which catalyze the reversible hydration of carbon dioxide (CO_2_) to bicarbonate (HCO_3_^−^) and a proton (H^+^). This simple reaction is essential for many physiological processes and it is carried out with the assistance of a bivalent ion (Zn^2+^ in vertebrates) located within the catalytic site of CAs. Human CAs (hCAs) belong to the α family, the first of the eight phylogenetically distinct classes identified so far, and 12 of the 15 isoforms expressed in humans are catalytically active [1].

While CAs are well-known and deeply investigated targets for designing inhibitors, there is scarce information on the mechanism of activation of these enzymes. Nevertheless, in the last few years, several lines of evidence have increased the interest in Carbonic Anhydrase Activators (CAAs). In fact, small polar molecules, such as amino acids and endogenous or synthetic amines, are able to increase the catalytic turnover of these enzymes [2]. Moreover, deficiencies of some human isoforms have been associated with the outbreak of pathological conditions, such as retinal degeneration, osteopetrosis, hyperammonemia, hyponatremia, hyperchlorhydrosis and cystic fibrosis [3,4,5,6,7,8,9], and CA activation has been shown to increase memory [10,11] and bone mineralization [12]. Therefore, various therapeutic applications can be envisaged for CAAs, provided these molecules are endowed with isoform selectivity.

**Figure 1:**
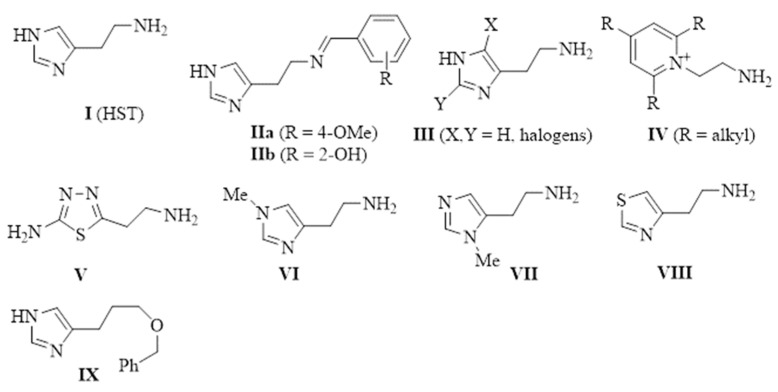
Chemical structure of CAAs reported in the literature.

Histamine (**I**, HST, Figure 1) is the prototypical CAA [2], and being one of the first such investigated derivatives, a multitude of structural modifications have been performed on its scaffold, with the aim to enhance its potency and/or selectivity. The X-ray crystallographic adduct of HST with hCA II [13] showed that the aliphatic amino moiety of the activator did not interact with the enzyme. Thus, this functional group was extensively modified for obtaining novel CAAs [2]. In particular, some interesting compounds were found in the imino series **II**, in which some compounds showed nanomolar K_A_ values and a marked preference for hCA VII [14]. The imidazole ring was also modified with the introduction of halogen atoms (**III**) [15] or it was substituted with pyridinium or thiadiazole moieties (in **IV [16]** and **V [17]**, respectively, Figure 1). More recently, HST receptor agonists **VI**–**IX** showed activating properties on several hCA isoforms with a higher potency compared to the parent compound **I** [18].

The crystal structure of HST bound within the hCA II active site showed a hydrogen bond network architecture involving the imidazole nitrogen atoms, supporting the hypothesis that HST improves the catalytic process through a proton shuttling mechanism [13], in which a pivotal role is played by His 64 (in the absence of activators). A similar pattern of interactions was also seen in the adduct of L-Histidine with hCA II [19]. Other amino acids such as D- and L-phenylalanine, or D-tryptophan were observed to bind at the entrance of the cavity, which delimits the catalytic site and stabilizes the active form of the enzyme through a network of H-bonds, involving this time, both the amino and carboxylic groups of the activator [20,21]. In the case of L-adrenaline, the H-bond network comprises the catechol and alcohol oxygen atoms too, suggesting that a basic nitrogen atom may not be essential [22]. In addition, there may be isoform-specific binding modes for these activators: for instance, in the X-ray structure of the L-histidine–CA II adduct, the imidazole ring is involved in the hydrogen bond network [19], whereas in the L-histidine–CA I adduct the same role was played by the carboxylic group of the activator [22]. In general, a common structural feature of CAAs is the presence of a flexible aliphatic tail bearing protonatable moieties with pKa values spanning between 6 and 8 [23].

**Figure 2:**
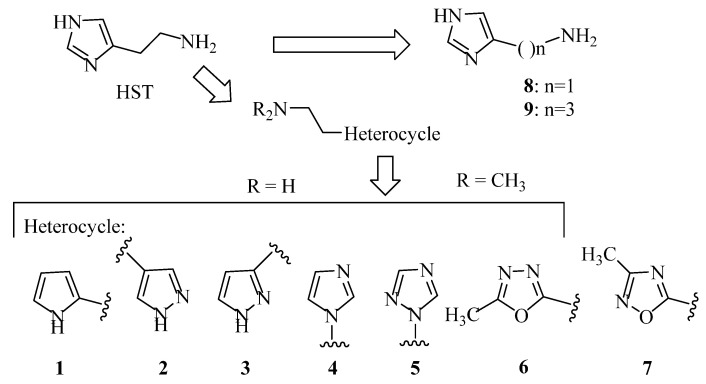
Chemical structure of histamine-related compounds described in this work.

In a previous paper, we showed that 2-amino-imidazolines structurally related to clonidine were able to activate the hCA isoform with K_A_ values in the micromolar range, suggesting that the imidazoline group could be a good bioisostere for the imidazole ring for novel types of CAAs [24]. As a continuation of this work, we here report the exploration of other 5-membered aromatic heterocycles: indeed, a new series of CAAs was synthesized, which was derived from the bioisosteric replacement of the HST imidazole moiety in order to infer SAR of this pharmacophore portion (compounds **1**–**7**, Figure 2). The new heterocycles keep the same ring size and shape of the imidazole ring, while for the heteroatoms, the basicity and the hydrogen bond donor/acceptor capability of the aromatic portions were varied [25]. Aiming to also change the proton-shuttling properties and compare the new compounds to HST, the primary amino group was maintained when possible. Additionally, the lower (**8**) and higher (**9**) homologs of HST were also prepared, in order to study the influence of chain length on SAR, and some modified derivatives of the most interesting compounds, **1** and **8**, were also obtained.

## 2. Results

### 2.1. Chemistry

Compounds **1** [26], **2** [27], **3** [27], **4** [28], **5** [29], **6** [30], **7** [31] and **8** [32], were prepared according to literature procedures. Attempts to prepare 2-(3-methyl-1,2,4-oxadiazol-5-yl)ethan-1-amine (the primary amine analog of **7**) according to Macor [31] failed due to the instability of the compound and only the elimination product, i.e., 3-methyl-5-vinyl-1,2,4-oxadiazole was obtained.

**Scheme 1:**
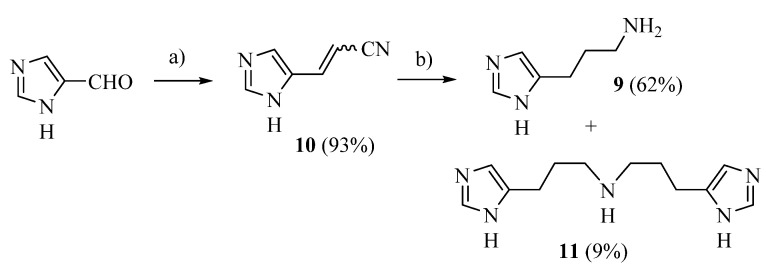
Synthesis of compounds **9** and **11**. Reagents: (a) (cyanomethyl)triphenylphosphonium chloride; (b) H_2_, Ni/Ra, EtOH/NH_3_.

The procedure of Sellier [33] was applied with some modification to prepare compound **9** (Scheme 1): commercially available 4-imidazolecarbaldehyde was reacted with (cyanomethyl)-triphenylphosphonium chloride [34] obtaining **10** [35] as a 6:4 cis/trans mixture, which was hydrogenated in a Parr apparatus using Raney nickel as a catalyst. The desired **9** was separated from the dimer **11** by means of flash chromatography.

**Scheme 2:**
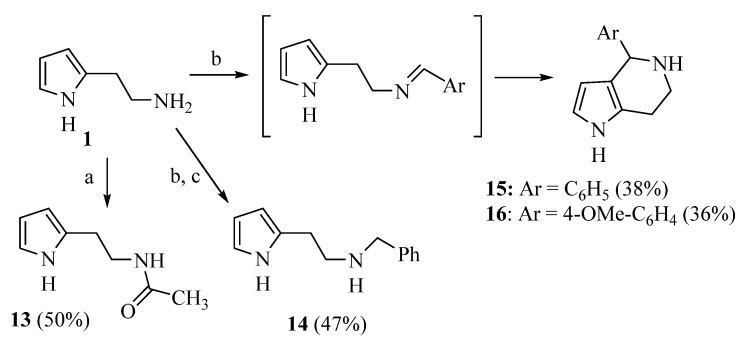
Synthesis of compounds **13**–**16**. Reagents: a. Acetic anhydride; b. ArCHO, 3Å molecular sieves, MeOH; c. NaBH_4_.

Due to the interesting activity of **1** on hCA I (see results), we decided to synthesize and test a small library of its analogs. Therefore, 2-(1-methyl-1H-pyrrol-2-yl)ethan-1-amine **12** (Table 1) was prepared from commercially available 1-methyl-1H-pyrrole-2-carbaldehyde following the literature procedure [36], whereas the acetyl (**13** [37]) and benzyl (**14**) derivatives were prepared from **1** through standard procedures (Scheme 2). We were also intrigued by the conversion of the primary amino group into a Schiff’s base since this modification confers in some instances isoform selectivity to HST, while generally enhancing the potency [14]. For this reason, p-anisaldehyde was chosen as the reactant together with benzaldehyde for comparison. Thus, **1** was reacted with benzaldehyde in MeOH using 3Å molecular sieves as the dehydrating agent. However, chromatographic separation on Al_2_O_3_ afforded only the cyclic derivative **15** as a racemate, probably deriving from a Pictet–Spengler condensation [38]. Similarly, the reaction of **1** with p-anisaldehyde afforded racemic **16**. Compound **15** has been previously synthesized by Herz and Tocker [37] from N-benzoyl-2-(2-pyrrole)-ethylamine through a Bischler–Napieralski reaction followed by reduction.

**Scheme 3:**
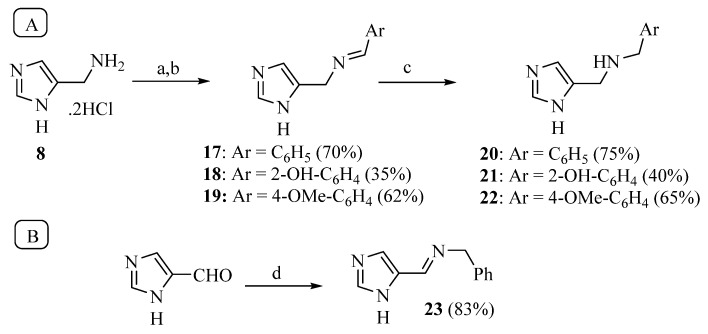
Synthesis of compounds **17**–**22** (**A**) and **23** (**B**). Reagents: a. KOH; b. ArCHO, 3Å molecular sieves, MeOH; c. NaBH4; d. MgSO4, benzylamine.

The synthesis of Schiff bases was thereafter performed from **8**, the lower homolog of HST (Scheme 3A). After neutralization of the hydrochloride salt with KOH, the reaction with benzaldehyde, salicylaldehyde and p-anisaldehyde smoothly afforded good yields of the corresponding imines **17**–**19**, which were then reduced with sodium borohydride to **20**–**22** [39,40]. The reaction of 4-imidazole-carbaldehyde with benzylamine yielded the Schiff base **23** [41] (Scheme 3B).

### 2.2. CA Activating Properties

The CO_2_ hydration activity catalyzed by different CA isoforms was measured by means of the stopped-flow method [42]. The potencies of the activators are expressed as K_A_ (activation constant, μM) values. For biological testing, the ubiquitous hCA I and hCA II; the mitochondrial hCA VA, on which HST is particularly potent [43]; hCA VII, mainly expressed in the brain [44,45], and hCA XIII, expressed in human reproductive organs [46,47], were chosen. The activation data of compounds **1**–**9** and **11**–**23** are reported in Table 1, with HST as the standard activator.

In general, none of the compounds were active on hCA II at the highest tested concentration, but as seen from data reported in the table, HST also has a low potency (K_A_ 125 μM) on this isoform. On the contrary, all the compounds were able to activate hCA VA, but none displayed the same potency of HST on this isoform. All compounds were effective activators of hCA VII too, with K_A_ values in the medium micromolar range.

#### 2.2.1. Activity on hCA I

The most sensitive isoform was hCA I, which was activated by all compounds with the exception of the oxygenated heterocyclic derivatives **6** and **7**, which were inactive on this isoform up to a 150 μM concentration. Among the closest structural analogs of HST, compounds **1**, **8** and **9** (K_A_ 2.16, 2.9 and 2.4 μM, respectively) were equipotent to the lead (HST, K_A_ 2.1 μM), whereas compounds **2**–**5** were 6–13 times less potent than the reference molecule. As mentioned in the introduction, the aliphatic amino group seems not to be essential for CA activation; this hypothesis is supported by the good potency displayed by alcohol **3a** (K_A_ 2.19 μM), an intermediate in the synthesis of **3**, which was found to be 13 times more potent than its amino analog (K_A_ 28.4 μM). A one carbon unit increase or reduction of the HST chain length did not affect activity, since compounds **9** (K_A_ 2.4 μM) and **8** (K_A_ 2.9 μM) were equipotent to the lead, while the dimer **11** (K_A_ 7.0 μM) was three times less active.

As far as the pyrrole derivatives **12–16** were concerned, the methylation of the endocyclic N atom (**12**, K_A_ 11.6 μM) or the acetylation of the primary amino group (**13**, K_A_ 5.6 μM) brought a five- and two-fold reduction of potency, respectively. On the contrary, the benzyl analog **14** (K_A_ 3.0 μM) and the tetrahydro-pyrrolo[3,2-c]pyridine **15** (K_A_ 3.7 μM) were almost equipotent to **1**. Finally, the insertion of a *para*-OMe group (**16**, K_A_ 5.7 μM) slightly decreased activity.

The conversion of the aliphatic amine into a Schiff base (compounds **17**–**19**, **23**) was detrimental for activity, since the K_A_ values were generally increased by 20–30 times compared to **8**. On the contrary, the reduction of imines **17**–**19** to secondary amines (**20**–**22**) restored the potency: compounds **20** and **21** were only two times less potent that **8**, while the potency of phenol **22** (K_A_ 0.9 μM) was three times higher compared to the primary amine **8**.

#### 2.2.2. Activity on hCA VA, VII and XIII and Selectivity

On hCA VA all the compounds were at least three orders of magnitude less potent than HST. The K_A_ values for all the synthesized derivatives ranged from 11.2 to 78.5 μM, meaning that all the structural modifications were detrimental to the potency. The narrow range of activity of our derivatives made it difficult to derive an appropriate SAR for this isoform. Some of the most active compounds (K_A_ values about 12 μM) were the HST homologs **8** and **9**, the dimer **11** and the benzyl derivatives **21** and **22**. The least active was the alcohol **3a** (K_A_ 78.5 μM).

On the contrary, the structural modifications reported in this work gave compounds with comparable or improved activity on hCA VII with respect to HST. All the heterocyclic analogs possessed K_A_ values similar to the lead, with the exception of the 1,2,4-oxadiazole **7** (K_A_ 12.1 μM), being three times more potent. Compounds **12–16**, with increased lipophilicity, were 3–4 times more active than the parent pyrrole **1**. The addition of a benzyl moiety on the lower homolog of HST did not substantially change the potency, as compounds **20**–**22** were equipotent to **8**, while Schiff bases **17**–**19** and **23** were 2–5 times less active. Contrary to what happened for hCA I, on hCA VII the amino derivative **3** was found to be more potent (five times) than alcohol **3a** (K_A_ 120 μM).

On hCA XIII the K_A_ of HST was in the low micromolar range, and none of our new compounds displayed a comparable or better potency. Moreover, compounds **1**–**7**, **13**, **15**, **16**, **22** and **23** were devoid of activity when tested up to a 100 μM concentration. The most active compounds were the HST homologs **8** and **9** (K_a_ about 29 μM) and the Schiff base **18** (K_A_ 33 μM).

As far as isoform selectivity is concerned, compounds **1**, **3a** and **22** showed a slight selectivity for hCA I. As a matter of fact, these compounds were inactive on hCA II and hCA XIII, while being more potent on hCA I than on hCA VA (14, 36 and 12 times, respectively) and hCA VII (21, 55 and 15 times, respectively).

## 3. Discussion

The bioisosteric replacement of the imidazole ring of HST with different 5-membered heterocycles (compounds **1**–**7**) gave contrasting results for obtaining novel CAAs. The most striking finding was that the activity on hCA II was lost for all compounds at doses up to 100–150 μM, meaning that every structural modification prevented the interaction with hCA II. Even if the potency of HST on this isoform is low, the lack of activity of its analogs was somewhat unexpected since previous works by Maccallini [48] and Provensi [18], showed how several other heterocycles (indazole, pyrazole, oxazole, thiazole or pyridine) maintained hCA II activating properties. On the contrary, the substitution of the imidazole ring with oxa-heterocycles, a modification detrimental to the potency on hCA I, improved the activity on hCA VII. Indeed, compound **7** was three times more potent than HST on the latter isoform. This modification deserves additional studies since, at present, it is difficult to clarify if this difference derives from the diverse heterocycle or from the introduction of the tertiary amino group. Unfortunately, the synthesis of the NH_2_ analog of **7** failed due to the instability of the oxadiazole derivatives. The inactivity of **9** on hCA II was unexpected too, since the low micromolar K_A_ value of compound **IX** (Figure 1; K_A_ 7.66 μM) [18], in which the NH_2_ group of **9** (K_A_ > 100 μM) was replaced by a benzyloxy group, suggests that the extension of the HST alkyl chain can be very well tolerated on hCA II.

As stated in the introduction, there is evidence that activators can change their binding mode according to the isoform. For hCA I, the most sensitive isoform to the compounds reported in this study, only the X-ray structure of the complex with L-histidine has been reported [15]. In addition, small activators such as **1**–**9**, can be accommodated in different ways within the binding site, and only a crystallographic study could cast light on their binding mode [49]. Unfortunately, all the attempts made so far to crystallize compound **1** with hCA I have failed. Some preliminary structure–activity relationships inferred from the results reported in Table 1 showed that the modifications made on pyrrole **1** had different consequences with respect to HST. In fact, the methylation of the heterocyclic nitrogen atom of **1** brought a five-fold increase of K_A_, while methylation of the equivalent position on HST (compound **VII**. Figure 1) did not affect activity [18]. The addition of an acetyl group on the exocyclic N atom of **1** (compound **13**) was detrimental (two-fold increase of K_A_) while the addition of small acyl groups on HST greatly improved the activity [50]. This evidence suggested a different binding mode of **1** with respect to HST on hCA II and, probably, on other isoforms too. A similar hypothesis can be proposed for compound **8**: the introduction of an imine moiety on HST (compounds **IIb** and **IIa**, Figure 1) improved the potency two and nine times, respectively on hCA I, and about three orders of magnitude on hCA VII [14]. On the contrary, the same modification on **8** (compounds **17**–**19** and **23**) was largely detrimental on hCA I and did not affect the potency on hCA VII. Since the same modifications performed on the HST scaffold or on our series of compounds led to different and sometimes opposite effects on activity, additional studies are needed to optimize the properties of these HST bioisosteres and, hopefully, discover compounds with a higher potency in order to obtain a complex useful for X-ray crystallography.

Some of the new compounds (**1**, **3a** and **22**) displayed an interesting selectivity for activating hCA I. This isoform is abundant in erythrocytes but is also found in other tissues (reviewed in [51]). In particular, the decrease in the expression and activity of CA I found in the erythrocytes of diabetic patients has been linked to insulin resistance [52]. Moreover, the downregulation of CA I in hepatocellular carcinoma with portal vein thrombus or in non-small cell lung cancer has been related to increased tumor cell motility promoting tumor growth and metastasis [53,54]. The discovery of selective CA I activators could help clarifying the role of this isoform in pathologies, possibly suggesting new therapeutic interventions.

## 4. Materials and Methods

### 4.1. Chemistry

#### 4.1.1. General Considerations

All melting points were taken on a Büchi apparatus and are uncorrected. NMR spectra were recorded on a Brucker Avance 400 spectrometer (400 MHz for ^1^H NMR, 100 MHz for ^13^C). ^1^H and ^13^C-NMR spectra of the final compounds are available in the Supplementary Material. Chromatographic separations were performed on a silica gel column by gravity chromatography (Kieselgel 40, 0.063–0.200 mm; Merck, Darmstadt, Germany) or flash chromatography (Kieselgel 40, 0.040–0.063 mm; Merck). Yields are given after purification, unless differently stated. When reactions were performed under anhydrous conditions, the mixtures were maintained under nitrogen. The purity of the final compounds was determined by an Agilent 1200 liquid chromatography system composed of an autosampler, binary pumps, column oven and diode-array detector (HPLC-DAD) operating in the UV range (200–400 nm). All compounds studied here were ≥95% HPLC-DAD pure. High-resolution mass spectrometry (HR-MS) analyses were performed with a Thermo Finnigan LTQ Orbitrap mass spectrometer equipped with an electrospray ionization source (ESI). Analyses were carried out in positive ion mode, monitoring protonated molecules, [M + H]^+^ species, and a proper dwell time acquisition was used to achieve 60,000 units of resolution at Full Width at Half Maximum (FWHM). The elemental composition of compounds was calculated on the basis of their measured accurate masses, accepting only results with an attribution error of less than 5 ppm and a non-integer RDB (double bond/ring equivalents) value, in order to consider only the protonated species [55]. The solvents used in HPLC-DAD and HRMS measures were acetonitrile (Chromasolv grade), and mQ water 18 MΩ cm. Compounds were named following IUPAC rules by means of ChemBioDraw Ultra 14.0.

#### 4.1.2. Compounds Prepared According to Literature Procedures

**2-(1H-pyrrol-2-yl)ethan-1-amine** (**1**) [26]. [^1^H]-NMR (CDCl_3_) δ: 1.82 (bs, 2H, NH_2_); 2.74 (t, *J* = 6.4 Hz, 2H, CH_2_Ar); 2.97 (t, *J* = 6.4 Hz, 2H, CH_2_N); 5.94 (s, 1H, H3); 6.13 (q, *J* = 2.8 Hz, 1H, H4); 6.68 (d, *J* = 2.8 Hz, 1H, H5); 9.01 (bs, 1H, NH pyrrole) ppm. [^13^C]-NMR (APT, CDCl_3_) δ: 30.72 (CH_2_Ar); 42.01 (CH_2_N); 105.49 (C3); 108.06 (C4); 116.49 (C5); 130.74 (C2) ppm. The compound was transformed into the oxalate salt, m.p. 144 °C dec.

**2-(1H-pyrazol-3-yl)ethan-1-amine** (**2**) [27]. [^1^H]-NMR (D_2_O) δ: 2.98 (t, *J* = 7.2 Hz, 2H, CH_2_Ar); 3.31 (t, *J* = 7.2 Hz, 2H, CH_2_N); 6.29 (d, *J* = 2.0 Hz, 1H, H4); 7.65 (d, *J* = 2.0 Hz, 1H, H5) ppm. [^13^C]-NMR (APT, D_2_O) δ: 22.72 (CH_2_Ar); 49.66 (CH_2_N); 105.18 (C4); 133.29 (C5); 165.43 (C3) ppm. The compound was transformed into the oxalate salt, m.p. 166 °C dec.

**2-(1H-pyrazol-4-yl)ethan-1-amine** (**3**) [27]. [^1^H]-NMR (CD_3_OD) δ: 2.67 (t, *J* = 7.2 Hz, 2H, CH_2_Ar); 2.86 (t, *J* = 7.2 Hz, 2H, CH_2_N); 7.47 (s, 2H, H3 and H5) ppm. [^13^C]-NMR (CD_3_OD) δ: 26.12 (CH_2_Ar); 42.03 (CH_2_N); 117.35 (C4); 132.71 (C3 and C5) ppm. M.p. 173 °C dec.

**2-(1H-pyrazol-4-yl)ethan-1-ol** (**3a**) [27]. Oil. [^1^H]-NMR (CD_3_OD) δ: 2.70 (t, *J* = 6.8 Hz, 2H, CH_2_Ar); 3.69 (t, *J* = 6.8 Hz, 2H, CH_2_O); 7.45 (s, 2H, H3 and H5) ppm. [^13^C]-NMR (CD_3_OD) δ: 27.11 (CH_2_Ar); 62.43 (CH_2_O); 117.26 (C4); 139.95 (C3 and C5) ppm.

**2-(1H-imidazol-1-yl)ethan-1-amine** (**4**) [24,28]. [^1^H]-NMR (CD_3_OD) δ: 2.98 (t, *J* = 6.2 Hz, 2H, CH_2_N); 4.07 (t, *J* = 6.2 Hz, 2H, CH_2_Ar); 6.98 (s, 1H, H4); 7.15 (s, 1H, H5); 7.67 (s, 1H, H2) ppm. [^13^C]-NMR (APT, CD_3_OD) δ: 41.83 (CH_2_N); 48.82 (CH_2_Ar); 119.31 (C5); 127.91 (C4); 137.31 (C2) ppm. The compound was transformed into the oxalate salt, m.p. 127 °C dec.

**2-(1H-1,2,4-triazol-1-yl)ethan-1-amine** (**5**) [29]. [^1^H]-NMR (CDCl_3_) δ: 1.73 (bs, 2H, NH_2_); 3.03 (t, *J* = 5.6 Hz, 2H, CH_2_N); 4.09 (t, *J* = 5.6 Hz, 2H, CH_2_Ar); 7.82 (s, 1H, H3); 8.04 (s, 1H, H5) ppm. [^13^C]-NMR (APT, CDCl_3_) δ: 41.29 (CH_2_N); 52.55 (CH_2_Ar); 143.55 (C5); 151.99 (C3) ppm. The compound was transformed into the oxalate salt, m.p. 194 °C dec.

**2-(5-methyl-1,3,4-oxadiazol-2-yl)ethan-1-amine** (**6**) [30]. A slight modification was performed in the last step: removal of the Boc protecting group was accomplished using trifluoroacetic acid. The base was obtained by partitioning between 4M NaOH and dichloromethane, and it was transformed into the oxalate salt, m.p.116–119 °C. [^1^H]-NMR (D_2_O) δ: 2.42 (s, 3H, CH_3_), 3.19 (t, *J* = 7.0 Hz, 2H, CH_2_), 3.36 (t, *J* = 7.0 Hz, 2H, CH_2_) ppm. [^13^C]-NMR (as oxalate salt, D_2_O) δ: 9.97 (CH_3_), 23.03 (CH_2_), 35.93 (CH_2_), 164.19 (C_q_), 164.74 (C_q_ oxa), 166.24 (C_q_) ppm.

**2-(3-methyl-1,2,4-oxadiazol-5-yl)ethan-1-amine** (**7**) [31]. [^1^H]-NMR (CDCl_3_) δ: 2.24 (s, 6H, N(CH_3_)_2_), 2.33 (s, 3H, ArCH_3_), 2.75 (t, *J* = 7.2 Hz, 2H, CH_2_), 2.98 (t, *J* = 7.2 Hz, 2H, CH_2_) ppm.

**(1H-imidazol-4-yl)methanamine (8)** [32]. M.p. 152–153 °C (lit. 239–242 °C). [^1^H]-NMR (MeOD) δ: 3.30 (s, 2H, CH_2_), 4.32 (s, 2H, CH_2_), 7.71 (s, 1H, H5), 8.99 (s, 1H, H2) ppm. [^13^C]-NMR (D_2_O) δ: 32.77 (CH_2_), 118.72 (CH, Im), 119.73 (C, Im), 134.63 (CH, Im) ppm. ESI-MS 98.1 [M + H]^+^.

**2-(1-methyl-1H-pyrrol-2-yl)ethan-1-amine** (**12**) [36]. [^1^H]-NMR (CDCl_3_) δ: 2.02 (bs, 2H, NH_2_), 2.71 (t, *J* = 6.8 Hz, 2H, NCH_2_) 2.95 (t, *J* = 6.8 Hz, 2H, CH_2_), 3.54 (s, 3H, NCH_3_), 5.92 (s, 1H, H3), 6.05 (t, *J* = 3.0 Hz, 1H, H4), 6.56 (s, 1H, H5) ppm. [^13^C]-NMR (APT, CDCl_3_) δ: 25.08 (CH_2_), 33.50 (NCH_3_), 38.83 (CH_2_), 106.85 (CH), 107.01 (CH), 122.23 (CH), 127.79 (C) ppm.

**N-(2-(1H-pyrrol-2-yl)ethyl)acetamide** (**13**) [37]. [^1^H]-NMR (CDCl_3_) δ: 1.90 (s, 3H, CH_3_CO), 2.77–2.80 (m, 2H, ArCH_2_), 3.42–3.46 (m, 2H, NCH_2_), 5.84 (bs, 1H, NH), 5.91 (s, 1H, H3), 6.10 (s, 1H, H4), 6.66 (s, 1H, H5), 8.72 (bs, 1H, NH) ppm. [^13^C]-NMR (APT, CDCl_3_) δ: 23.30 (CH_3_), 27.95 (CH_2_), 39.39 (CH_2_), 105.92 (CH), 108.36 (CH), 117.16 (CH), 128.86 (C), 170.70 (CO) ppm.

**N-benzyl-1-(1H-imidazol-4-yl)methanimine** (**23**) [41] M.p. 170–172 °C. [^1^H]-NMR (MeOD) δ: 4.72 (s, 2H, CH_2_), 7.18–7.37 (m, 5H, Ph), 7.55 (s, 1H, H5), 7.76 (s, 1H, H2), 8.37 (s, 1H, HC=N) ppm. [^13^C]-NMR (MeOD) δ: 64.14 (CH_2_), 126.84 (CH), 127.86 (CH), 128.18 (CH), 137.1 (C), 138.8 (CH=N) ppm.

#### 4.1.3. 3-(1H-imidazol-4-yl)propan-1-amine (**9**)

DBU (0.336 g, 2.21 mmol) was added dropwise to a suspension of 1H-imidazole-4-carbaldehyde (163.35 mg, 1.7 mmol, 1 eq) and (cyanomethyl)triphenylphosphonium chloride (0.634 g, 1.87 mmol, 1.1 eq) in toluene dry (15 mL) under nitrogen. The mixture was refluxed for 4 h; after removal of the solvent, the residue was vigorously stirred with H_2_O (50 mL) and the precipitate (Ph_3_PO) was filtered off. Extraction with ethyl acetate, anhydrification (Na_2_SO_4_) and removal of the solvent under vacuum gave a residue, 3-(1H-imidazol-5-yl)acrylonitrile **10** as a 6:4 cis/trans mixture (yield: 93.4%) impure with Ph_3_PO (7%). [^1^H]-NMR (MeOD) δ: 5.42 (d, *J* = 11.9 Hz, 0.6H, =CHCN cis), 5.94 (d, *J* = 16.3 Hz, 0.4H, =CHCN trans), 7.18 (d, *J* = 11.9 Hz, 0.6H, ArCH = cis), 7.38 (d, *J* = 16.0 Hz, 0.4H, ArCH = trans), 7.40 (s, 0.4H, H5 trans), 7.76 (s, 1H, H2 trans + H5 cis), 7.81 (s, 1H, H2 cis) ppm. Compound **10** was dissolved in abs EtOH (5 mL); abs EtOH saturated with NH_3_ (5 mL) was added followed by 0.5 mL of Raney nickel 2400 slurry in water, and the mixture was hydrogenated in a Parr apparatus at 65 psi overnight. Filtration over celite and removal of the solvent gave a residue, which was purified by flash chromatography on Al_2_O_3_, yielding **11** (first eluted compound, 9% yield) and **9** (second eluted compound, 62% yield).

**9** [33] (as free base) [^1^H]-NMR (MeOD) δ: 1.76 (p, *J* = 7.2 Hz, 2H, C–CH_2_–C), 2.59 (t, *J* = 7.2 Hz, 2H, CH_2_), 2.63 (t, *J* = 7.2 Hz, 2H, CH_2_), 6.75 (s, 1H, H5), 7.52 (s, 1H, H2) ppm. ESI-MS: 126.1 [M + H]^+^.

**11** ESI-HRMS (*m*/*z*) calculated for [M + H]^+^ ion species C_12_H_20_N_5_: 234.1719; found 234.1721. **11**.3HCl: [^1^H]-NMR (D_2_O) δ: 1.81–1.96 (m, 2H, C–CH_2_–C), 2.64 (t, *J* = 6.4 Hz, 2H, CH_2_), 2.92 (t, *J* = 6.9 Hz, 2H, CH_2_), 7.04 (s, 1H, H5), 8.24 (s, 1H, H2) ppm. [^13^C]-NMR (APT, MeOD) δ 21.23 (CH_2_), 24.86 (CH_2_), 46.87 (CH_2_), 116.14 (CH), 132.45 (C), 133.74 (CH) ppm.

#### 4.1.4. *N*-benzyl-2-(1H-pyrrol-2-yl)ethan-1-amine (**14**)

A mixture of **1** (60 mg, 0.54 mmol) and benzaldehyde (57 mg, 0.54 mmol) in dry MeOH (2.6 mL) was stirred at room temperature overnight with 3Å molecular sieves, which were removed before the addition of NaBH_4_ (204.1 mg, 5.4 mmol, 10 eq) at 0 °C. After 1 h stirring at room temperature, the reaction was quenched with ice and the volatiles were evaporated; the residue was dissolved in CH_2_Cl_2_ (25 mL) and washed with sat NaHCO_3_ (5 mL) and with brine (5 mL). Drying (Na_2_SO_4_) gave a residue purified through flash chromatography (CH_2_Cl_2_/MeOH/NH_3_ 95:5:0.5 as eluent) obtaining **14** (47% yield) and **15** (37%, see below). [^1^H]-NMR (CDCl_3_) δ: 2.82 (s, 2H, CH_2_), 2.92 (s, 2H, CH_2_), 3.73 (s, 2H, PhCH_2_), 4.68 (bs, 1H, NH), 5.83 (s, 1H, H3), 5.98 (s, 1H, H4), 6.60 (s, 1H, H5), 7.23–7.32 (m, 5H, Ph), 9.22 (bs, 1H, NH) ppm. [^13^C]-NMR (APT, MeOD) δ 26.9 (CH_2_), 48.33 (CH_2_), 52.8 (CH_2_), 104.9 (CH), 107.1 (CH), 116.3 (CH), 126.9 (CH), 128.0 (CH), 128.1 (CH), 128.9 (C), 138.8 (C) ppm. ESI-HRMS (*m*/*z*) calculated for [M + H]^+^ ion species C_13_H_17_N_2_: 201.1386; found 201.1384.

#### 4.1.5. 4-phenyl-4,5,6,7-tetrahydro-1H-pyrrolo[3,2-c]pyridine (**15**)

A mixture of **1** (90 mg, 0.82 mmol) and benzaldehyde (87 mg, 0.82 mmol) in dry MeOH (4 mL) was stirred at room temperature overnight with 3Å molecular sieves. Filtration and removal of the solvent under vacuum gave a residue which was purified by flash chromatography (CH_2_Cl_2_/MeOH/NH_3_ 95:5:0.5 as eluent), yielding the title compound in a 38% yield as a solid, m.p. 157–8 °C (lit. 212 °C) [37].

[^1^H]-NMR (MeOD) δ: 2.58–2.64 (m, 1H), 2.73–2.81(m, 1H), 2.91–2.98 (m, 1H), 3.15–3.20 (m, 1H), 4.90 (s, 1H, CHPh), 5.52 (d, *J* = 2.7 Hz, 1H), 6.51 (d, *J* = 2.7 Hz, 1H), 7.19–7.29 (m, 5H, Ph) ppn. [^13^C]-NMR (APT, MeOD) δ 22.67 (CH_2_), 41.79 (CH_2_), 58.37 (CH), 104.84 (CH), 115.71 (CH), 117.26 (CH), 125.14 (CH), 127.01 (CH), 127.82 (CH), 128.25 (CH), 143.80 (C) ppm ESI-MS (*m*/*z*) 199.1 [M + H]^+^.

#### 4.1.6. 4-(4-methoxyphenyl)-4,5,6,7-tetrahydro-1H-pyrrolo[3,2-c]pyridine (**16**)

The procedure used to obtain **15** was applied, starting with **1** (60 mg) and p-anisaldehyde (66.2 μL). Compound **16** was obtained in a 36% yield after purification by means of flash chromatography on Al_2_O_3_ using CH_2_Cl_2_/MeOH 95:5 as the eluent. [^1^H]-NMR (MeOD) δ: 2.59–2.69 (m, 1H), 2.75–2.86 (m, 1H), 2.95–3.04 (m, 1H), 3.17–3.25 (m, 1H), 3.74 (s, 3H, OMe), 4.94 (s, 1H), 5.53 (d, *J* = 2.7 Hz, 1H), 6.54 (d, *J* = 2.7 Hz, 1H), 6.83 (d, *J* = 8.6 Hz, 2H, Ph), 7.20 (d, *J* = 8.6 Hz, 2H, Ph) ppm. [^13^C]-NMR (APT, MeOD) δ: 22.23 (CH_2_), 41.58 (CH_2_), 54.31 (CH), 57.79 (OCH_3_), 104.88 (CH), 113.24 (CH), 115.95 (CH), 116.82 (C), 124.79 (C), 129.48 (CH), 134.84 (C), 159.35 (C) ppm. ESI-HRMS (*m*/*z*) calculated for [M + H]^+^ ion species C_14_H_17_N_2_O: 229.1335; found 229.1333.

#### 4.1.7. General Procedure for the Synthesis of Schiff Bases (**17**–**19**)

A solution of **8**.2HCl (60–80 mg) in dry MeOH (3–4 mL) was treated with KOH (2 eq). After 20 min stirring at room temperature, the suitable aldehyde (0.9 eq) was added and the mixture was stirred at room temperature overnight with 3Å molecular sieves. Filtration and removal of the solvent gave a residue which was treated with ethyl acetate; the insoluble material was filtered off, and the solvent was evaporated yielding the desired Schiff base. With this procedure the following compounds were prepared:

**N-((1H-imidazol-4-yl)methyl)-1-phenylmethanimine** (**17**) Glassy solid, yield: 69.7% [^1^H]-NMR (MeOD) δ: 4.73 (s, 2H, CH_2_Ar), 7.01 (s, 1H, CH, H5), 7.39–7.44 (m, 3H, Ph), 7.64 (s, 1H, H2), 7.73–7.75 (m, 2H, Ph), 8.43 (s, 1H, CH=N) ppm. [^13^C]-NMR (APT, MeOD) δ: 56.31 (CH_2_), 116.8 (CH, C5), 128.09 (CH, Ph), 128.41 (CH, Ph), 130.88 (CH, Ph), 135.23 (C), 135.31 (CH, C2), 135.66 (C), 163.96 (CH=N) ppm. Anal.: calculated for C_11_H_11_N_3_ (%): C, 71.33; H, 5.99; N, 22.69. Found: C, 70.99; H, 5.65; N, 22.91

**N-((1H-imidazol-4-yl)methyl)-1-(4-methoxyphenyl)methanimine** (**18**) Glassy solid, yield: 35%. 3.82 (s, 3H, OCH_3_), 4.69 (s, 2H, CH_2_), 6.96 (d, *J* = 8.7 Hz, 2H, Ph), 7.00 (s, 1H, H5), 7.63 (s, 1H, H2) 7.69 (d, *J* = 8.7 Hz, 2H, Ph), 8.35 (s, 1H, HC=N) ppm. [^13^C]-NMR (APT, MeOD) δ: 54.48 (OCH_3_), 56.15 (CH_2_), 113.77 (CH, Ph), 114.10 (CH, C5), 128.32 (C), 129.84 (CH, Ph), 135.24 (CH, C2), 162.30 (C), 163.47 (CH, HC=N) ppm. Anal.: calculated for C_12_H_13_N_3_O (%): C, 66.96%; H, 6.09%; N, 19.52%. Found C, 66.61%; H, 5.79%; N, 19.82%

**2-((((1H-imidazol-4-yl)methyl)imino)methyl)phenol** (**19**) Solid, m.p. 130–133 °C, 62% yield. [^1^H]-NMR (MeOD) δ: 4.73 (s, CH_2_), -), 6.82–6.84 (m, 2H), 7.03 (s, 1H, H5), 7.28 (t, *J* = 7.8 Hz, 1H), 7.31 (d, *J* = 7.7 Hz, 1H) 7.66 (s, 1H, H2), 8.48 (s, 1H, HC=N) ppm. [^13^C]-NMR (APT, MeOD) δ: 54.07 (CH_2_), 116.34 (CH, C5), 116.70 (CH), 118.03 (CH), 118.63 (C), 131.60 (CH), 132.37 (CH), 135.27 (CH, C2), 162.04 (C), 166.11 (CH, CH=N) ppm. Anal.: calculated for C_11_H_11_N_3_O (%): C, 65.66; H, 5.51; N, 20.88. Found C, 65.93; H, 5.30; N, 20.99.

#### 4.1.8. General Procedure for the Reduction of Schiff Bases to Amines **20**–**22**

The suitable Schiff base (50–60 mg) was dissolved in anhydrous MeOH (4–5 mL) and NaBH_4_ (10 eq) was added portionwise at 0 °C. The mixture was stirred for 1 h at room temperature, then ice was added. After removal of the solvent under vacuum, the residue was partitioned between ethyl acetate and water. Drying (Na_2_SO_4_) and removal of the solvent gave the desired compound. By this procedure, the following compounds were prepared:

**N-((1H-imidazol-4-yl)methyl)-1-phenylmethanamine (20)** [39]. Solid, m.p.197–198 °C (lit. 103–104 °C); 75% yield. [^1^H]-NMR (MeOD) δ: 3.74 (s, 2H), 3.76 (s, 2H), 6.09 (bs, 2H, NH), 6.80 (s, 1H, CH, H5), 7.16–7.31 (m, 5H, Ph), 7.43 (s, 1H, CH, H2) ppm.

**N-((1H-imidazol-4-yl)methyl)-1-(4-methoxyphenyl)methanamine** (**21**) [39]. Purification by flash chromatography (DCM/MeOH/NH_3_ 90:10:1 as eluent); oil, 40% yield. [^1^H]-NMR (MeOD) δ 3.65 (s, 2H, CH_2_), 3.68 (s, 2H, CH_2_), 3.73 (s, 3H, OCH_3_), 6.84 (d, *J* = 8.6 Hz, 2H, Ph), 6.96 (s, 1H, H5), 7.21 (d, *J* = 8.6 Hz, 2H, Ph), 7.60 (s, 1H, H2) ppm. [^13^C]-NMR (APT, MeOD) δ: 44.05 (CH_2_), 51.58 (CH_2_), 54.28 (CH_3_, OCH_3_), 113.29 (CH, C5), 113.49 (CH), 129.51 (CH), 130.81 (C), 135.03 (CH, C2), 159.06 (C) ppm. ESI-MS 218.1 [M + H]^+^.

**2-((((1H-imidazol-5-yl)methyl)amino)methyl)phenol** (**22**) Purification by flash chromatography (DCM/MeOH/NH_3_ 85:15:1.5 as eluent); oil, 65% yield. [^1^H]-NMR (MeOD) δ: 3.71 (s, 2H, CH_2_), 3.82 (s, 2H, CH_2_), 6.70–6.74 (m, 2H, Ph), 6.95 (s, 1H, H5), 7.02–7.08 (m, 2H, Ph), 7.59 (s, 1H, H2) ppm. [^13^C]-NMR (APT, MeOD) δ: 43.82 (CH_2_), 49.33 (CH_2_), 115.14 (CH), 115.83 (CH, C5), 118.89 (CH), 123.55 (C), 128.23 (CH), 129.17 (CH), 135.11 (CH, C2), 156.88 (C) ppm. ESI-HRMS (*m*/*z*) calculated for [M + H]^+^ ion species C_11_H_14_N_3_O: 204.1131; found 204.1130.

### 4.2. CA Activation

An Sx.18Mv-R Applied Photophysics (Oxford, UK) stopped-flow instrument was used to assay the catalytic activity of various CA isozymes for the CO_2_ hydration reaction [42]. Phenol red (at a concentration of 0.2 mM) was used as the indicator, working at the absorbance maximum of 557 nm, with 10 mM Hepes (pH 7.5, for α-CAs) as the buffer, and 0.1 M NaClO_4_ for maintaining a constant ionic strength. The CA-catalyzed CO_2_ hydration reaction was followed for a period of 10 s at 25 °C. The CO_2_ concentrations used for the determination of the kinetic parameters and inhibition constants ranged from 1.7 to 17 mM. For each activator at least six traces of the initial 5–10% of the reaction were used for determining the initial velocity. The uncatalyzed rates were determined in the same manner and subtracted from the total observed rates. Stock solutions of activators (at 0.1 mM) were prepared in distilled-deionized water and dilutions up to 1 nM were made thereafter with the assay buffer. Enzyme and activator solutions were preincubated together for 15 min prior to assay, in order to allow for the formation of the enzyme–activator complexes. The activation constant (K_A_), defined similarly with the inhibition constant K_I_, was obtained by considering the classical Michaelis–Menten equation (Equation (1)), which was fitted by nonlinear least squares using PRISM 3:

v = vmax/{1 + (K_M_/[S])(1 + [A]_f_/K_A_)}, (1)

where [A]_f_ is the free concentration of the activator.

Working at substrate concentrations considerably lower than K_M_ ([S] << K_M_), and considering that [A]_f_ can be represented in the form of the total concentration of the enzyme ([E]_t_) and activator ([A]_t_), the obtained competitive steady-state equation for determining the activation constant is given by Equation (2):

v = v_0_.K_A_/{K_A_ + ([A]_t_ − 0.5{([A]_t_ + [E]_t_ + K_A_) − ([A]_t_ + [E]_t_ + K_A_)^2^ − 4[A]_t_.[E]_t_)^1/2^}}, (2)

where v_0_ represents the initial velocity of the enzyme-catalyzed reaction in the absence of the activator.

## 5. Conclusions

In conclusion, in this work we reported the synthesis and the hCA activating properties of a new series of histamine-related molecules. In particular, among the most interesting compounds we mention are the cyclic derivatives **15** and **16**, which represent rigid analogs of **1** and **14**, and showed K_A_ values in the low (hCA I) and medium (hCA VA and VII) micromolar range. These molecules represent new chemotypes in the field of CA activators, deserving further investigation. Moreover, the compounds reported in this paper showed a general preference for hCA I. Notably, compounds **1**, **3a** and **22** displayed the best selectivity ratio for this isoform compared to hCA II, VA, VII and XIII. These substances can represent lead molecules, the optimization of which could possibly result in hCA I selective activators. Owing to the wide distribution of this isoform, such molecules could be useful as tools to study the role of hCA I in physiological and pathological conditions.

## Figures and Tables

**Table 1:** CA activation of isoforms hCA I, II, VA, VII and XIII with compounds **1**–**9**, **11**–**23**, by a stopped-flow CO_2_ hydrase assay. Histamine (HST) was used as the standard activator.

Cmp (Salt) ^a^	Structure	K_A_ (μM) ^b^				
		hCA I	hCA II	hCA VA	hCA VII	hCA XIII
HST	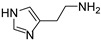	2.10	125	0.010	37.5	4.6
**1** (oxalate)	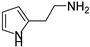	2.16	>150	29.8	44.6	>100
**2** (oxalate)	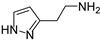	11.6	>150	37.9	32.8	>100
**3**	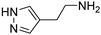	28.4	>150	51.0	23.7	>100
**3a**	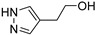	2.19	>150	78.5	120	>100
**4**	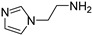	13.5	>150	42.7	25.4	>100
**5**	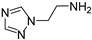	9.84	>150	24.6	35.5	>100
**6** (oxalate)	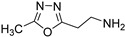	>150	>150	21.7	23.0	>100
**7** (HCl)	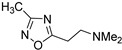	>150	>150	28.6	12.1	>100
**8**.2HCl	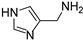	2.9	>100	12.5	13.2	29.0
**9**.2HCl	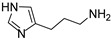	2.4	>100	12.5	10.8	28.2
**11**.3HCl	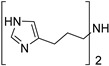	7.0	>100	12.9	11.5	84.8
**12**	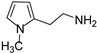	11.6	>100	34.9	13.8	96.4
**13**	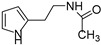	5.6	>100	24.8	10.8	>100
**14**	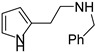	3.0	>100	14.0	12.5	91.1
**15**	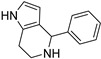	3.7	>100	20.6	11.1	>100
**16**	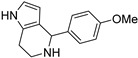	5.7	>100	19.1	14.3	>100
**17**	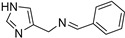	59.1	>100	15.2	38.6	64.4
**18**	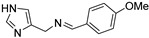	56.1	>100	16.0	22.1	33.0
**19**	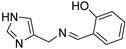	93.5	>100	13.3	35.4	57.3
**20**	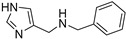	5.8	>100	27.2	9.7	46.0
**21**	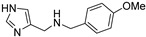	4.9	>100	11.9	18.3	73.0
**22**	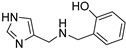	0.9	>100	11.2	13.2	>100
**23**	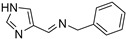	86.0	>100	18.8	74.6	>100

^a^ All compounds were tested as bases, with the exception of **1**, **2**, **6**–**9** and **11** (salts as indicated). ^b^ Mean from 3 different determinations (errors in the range of 5–10% of the reported values, data not shown).

## Data Availability

Not applicable.

## Supplementary Materials

The following are available online: ^1^H and ^13^C-NMR spectra of the final compounds.

## Sample Availability

Samples not available.
