# Supplementary material for: New Histamine-Related Five-Membered N-Heterocycle Derivatives as Carbonic Anhydrase I Activators
Source: Molecules. 2022 Jan 15;27(2):545. doi: 10.3390/molecules27020545 (PMC8779960; doi:10.3390/molecules27020545)
Supplement: Supplementary file 1 [file molecules-27-00545-s001.zip › molecules-1543065-supplementary.pdf]

Supplementary Materials: New Histamine-Related Five-Membered N-Heterocycle Derivatives as Carbonic Anhydrase I Activators

Niccolò Chiaramonte, Alessio Gabellini, Andrea Angeli, Gianluca Bartolucci, Laura Braconi, Silvia Dei, Elisabetta Teodori, Claudiu T. Supuran and Maria Novella Romanelli

1H and 13C-NMR spectra of the final compounds

^1H-NMR ($CDCl_3$)

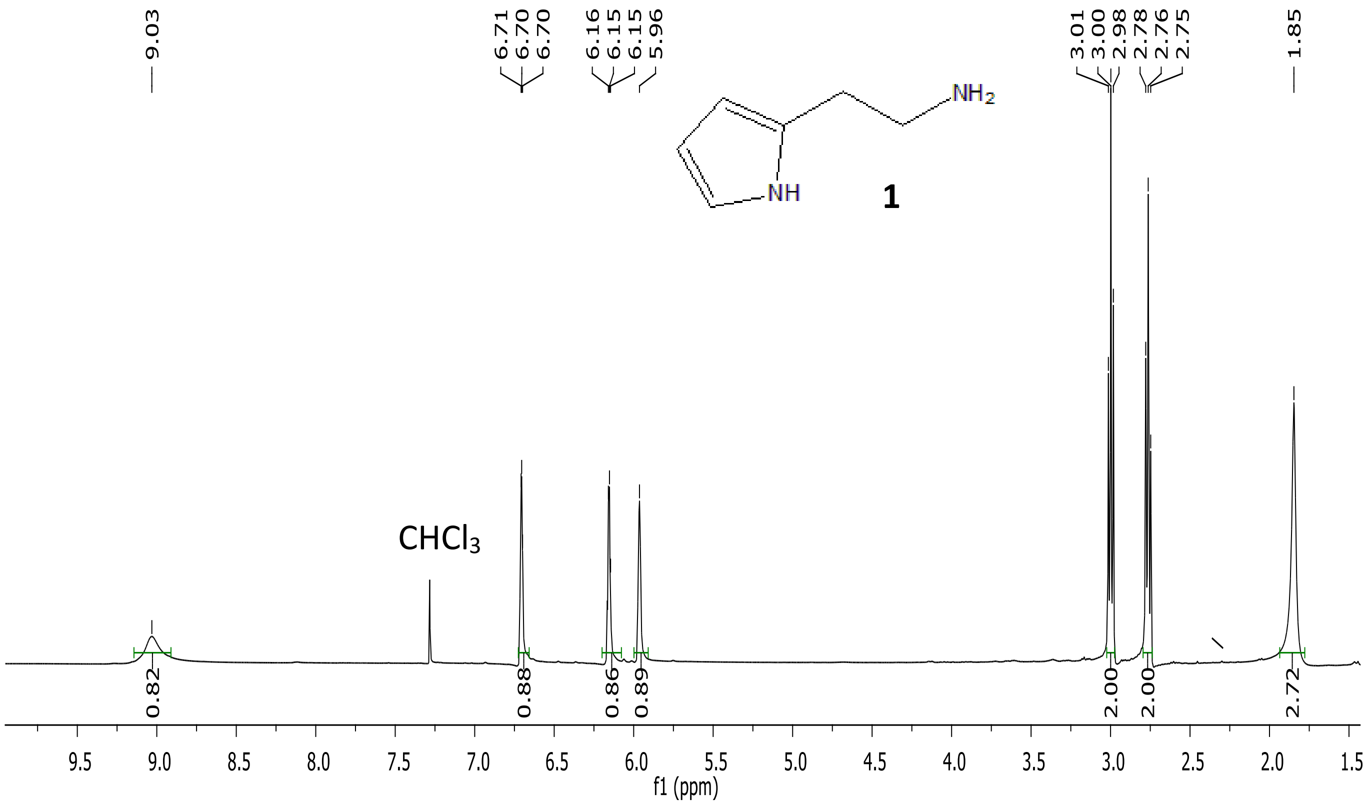

^{13}C-NMR
(APT, $CDCl_3$)

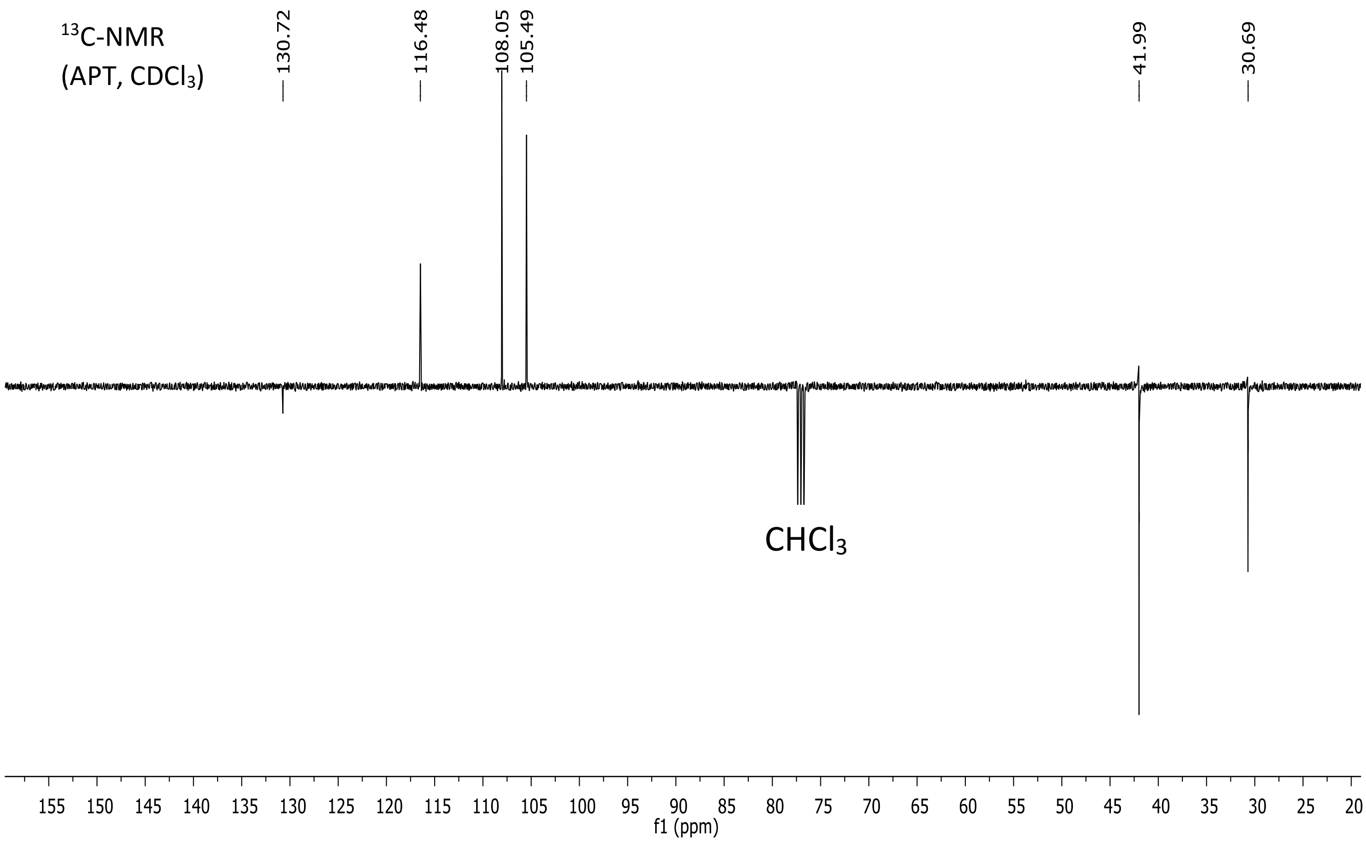

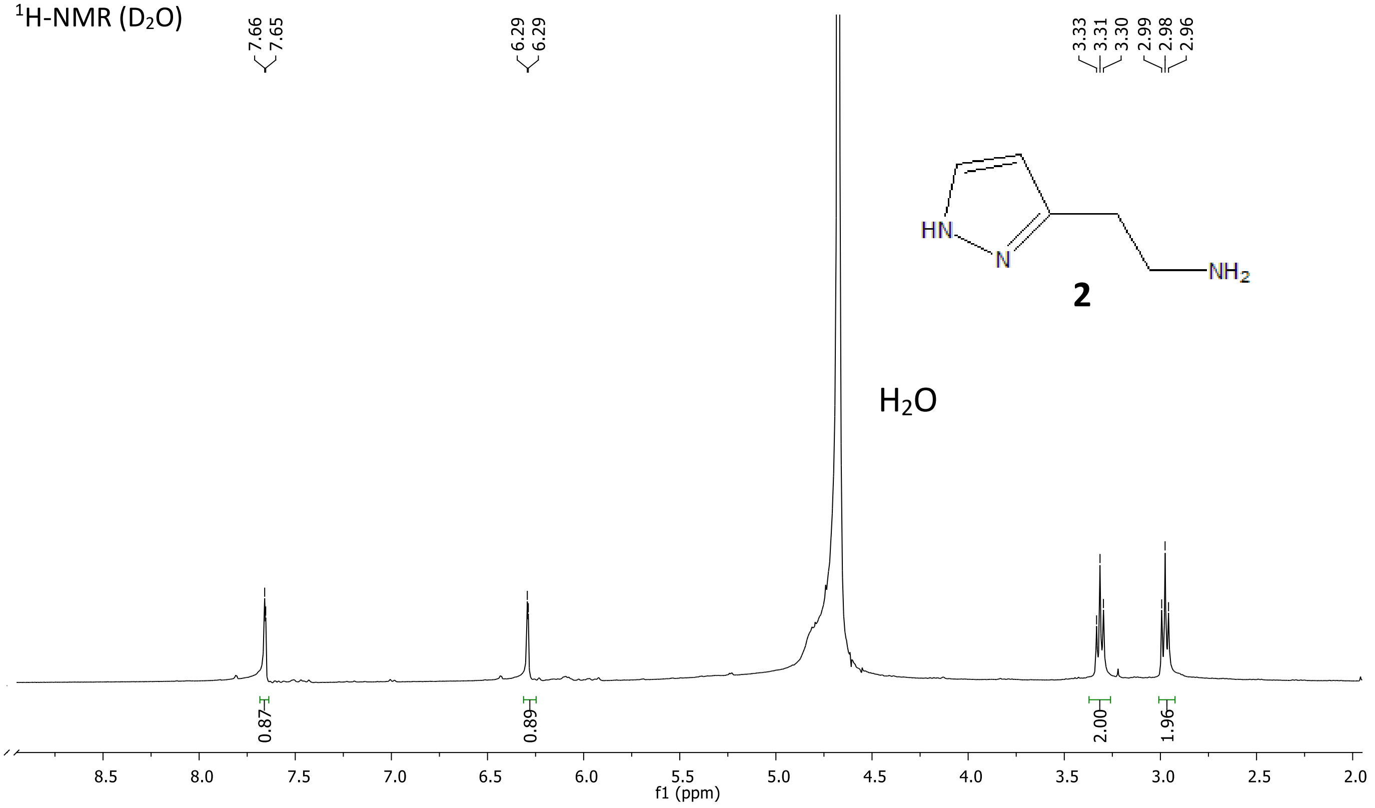

^{1}H-NMR (D_2O)
7.66
7.65
6.29
6.29
3.33
3.31
3.30
2.99
2.98
2.96
HN
N
NH_2
2
H_2O
0.87
0.89
2.00
1.96
8.5
8.0
7.5
7.0
6.5
6.0
5.5
5.0
4.5
4.0
3.5
3.0
2.5
2.0
f1 (ppm)

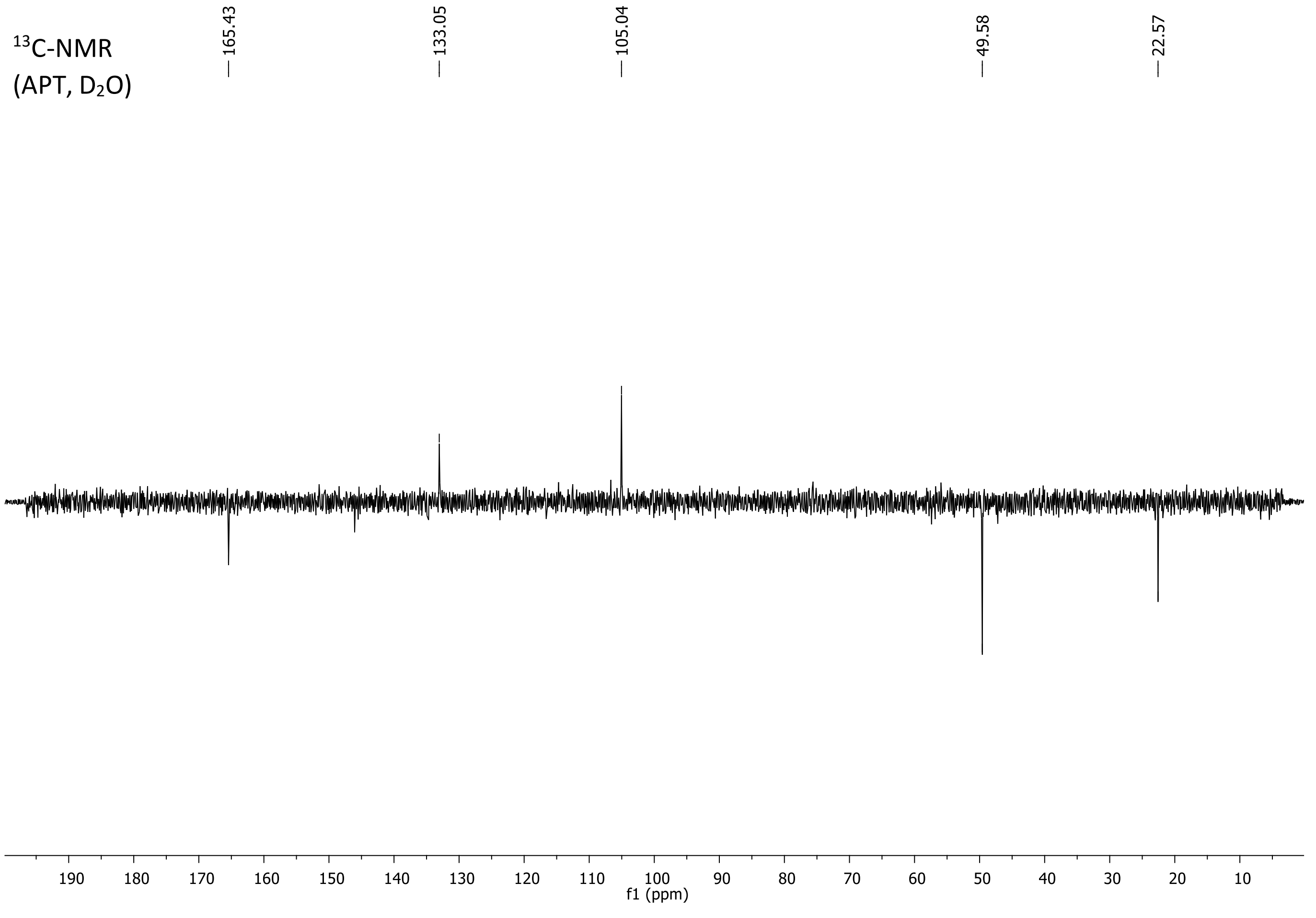

^{13}C-NMR
(APT, D_2O)
165.43
133.05
105.04
49.58
22.57
190
180
170
160
150
140
130
120
110
100
90
80
70
60
50
40
30
20
10
f1 (ppm)

^{1}H-NMR (CD_3OD)

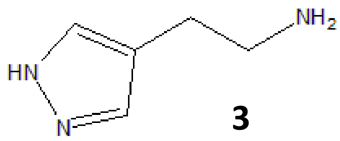

3

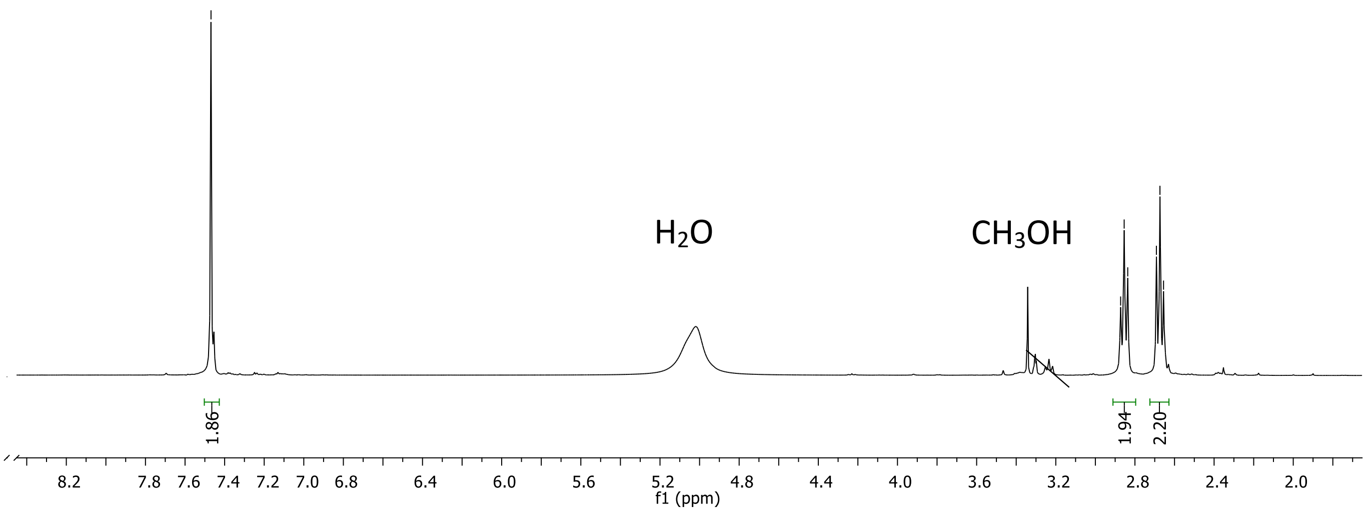

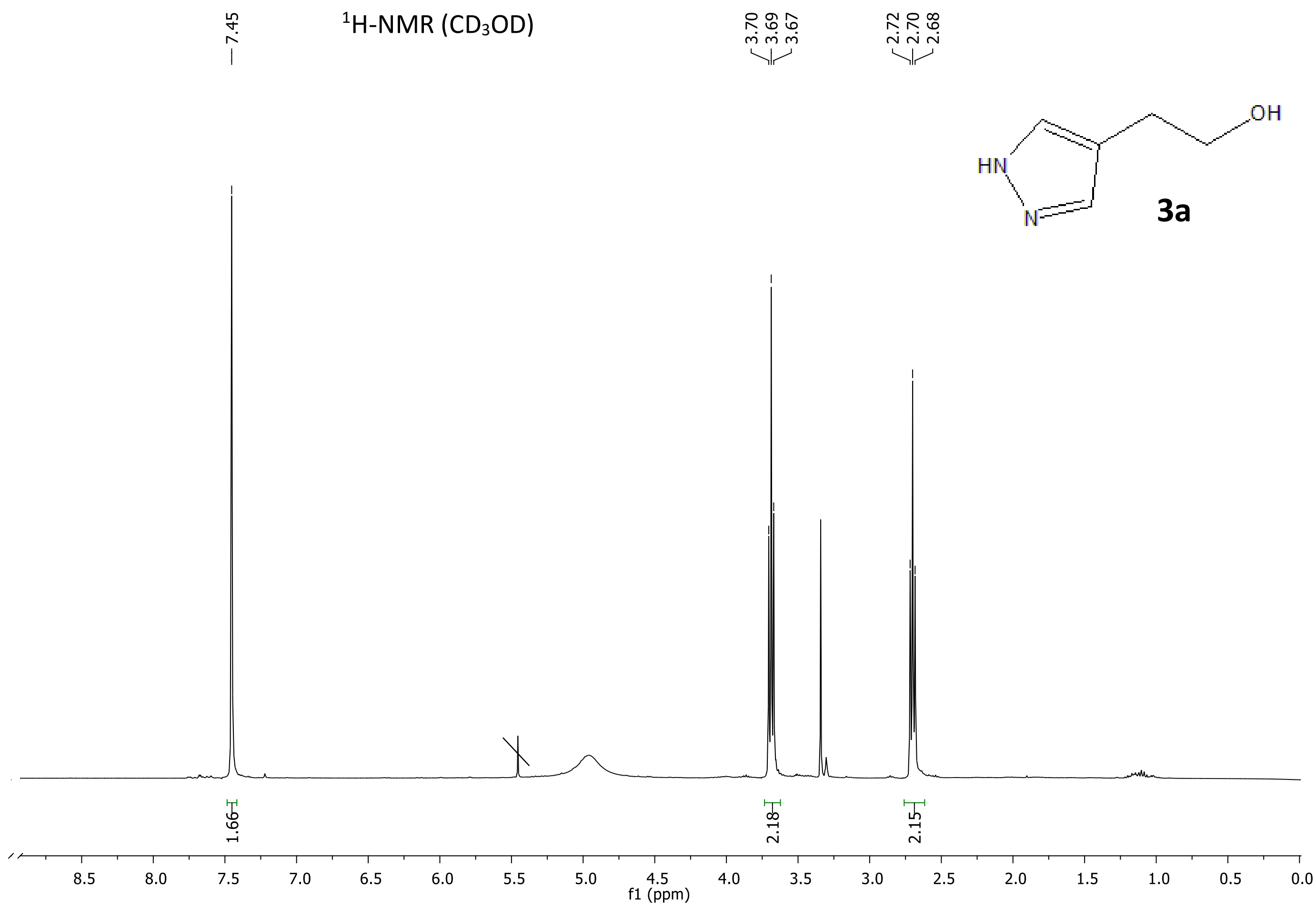

1H-NMR (CD3OD)
7.45
3.70
3.69
3.67
2.72
2.70
2.68
HN
N
OH
3a
1.66
2.18
2.15
8.5 8.0 7.5 7.0 6.5 6.0 5.5 5.0 4.5 4.0 3.5 3.0 2.5 2.0 1.5 1.0 0.5 0.0
f1 (ppm)

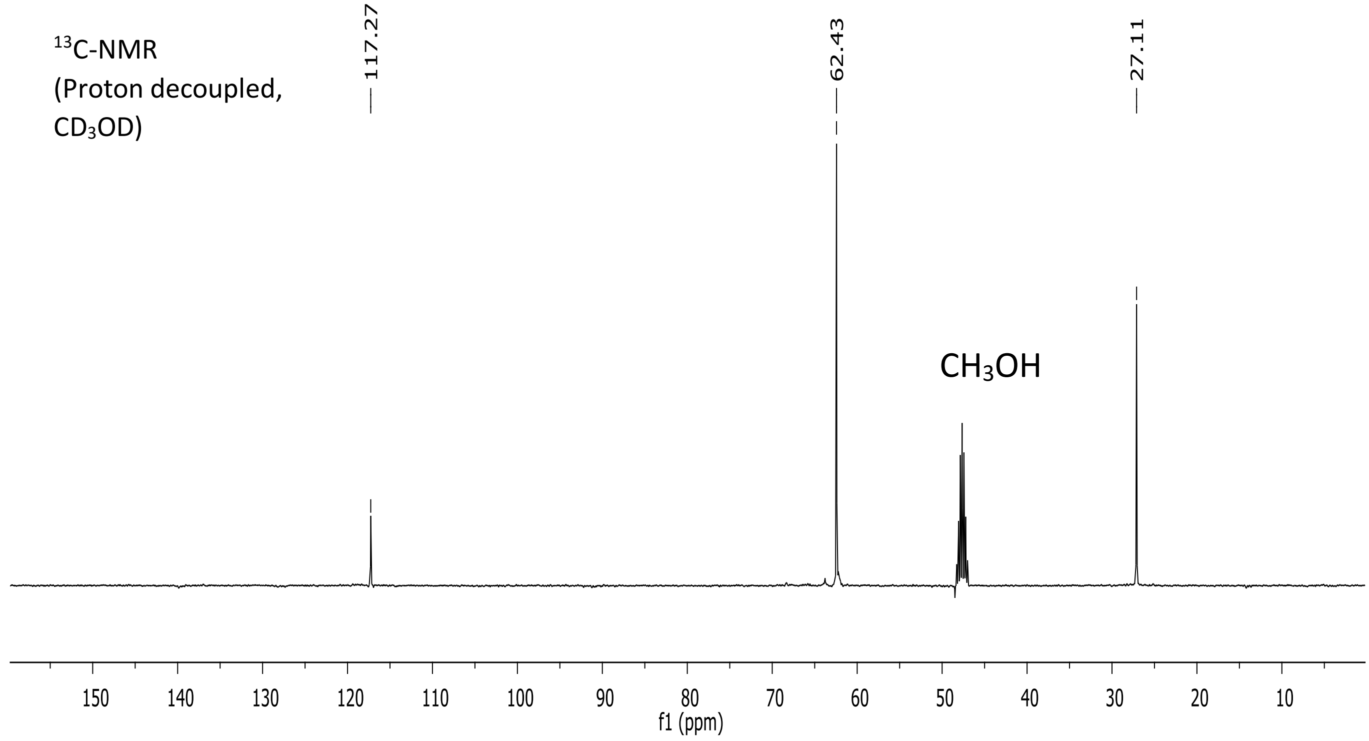

13C-NMR
(Proton decoupled,
CD3OD)
117.27
62.43
27.11
CH3OH
150 140 130 120 110 100 90 80 70 60 50 40 30 20 10
f1 (ppm)

^{1}H-NMR (CD_3OD)

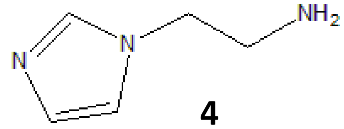

4

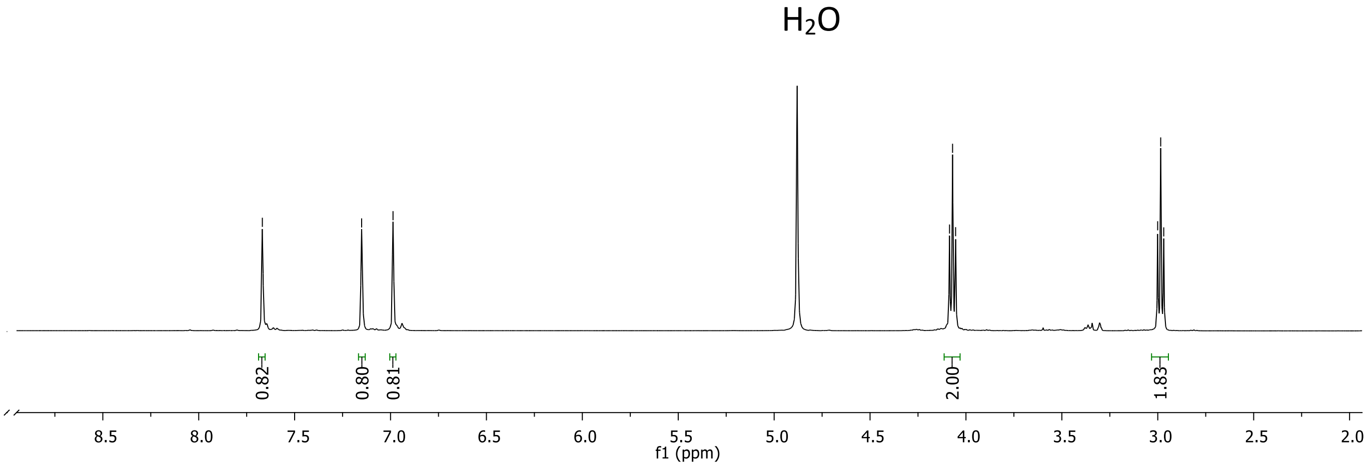

^{13}C-NMR
(APT, CD_3OD)

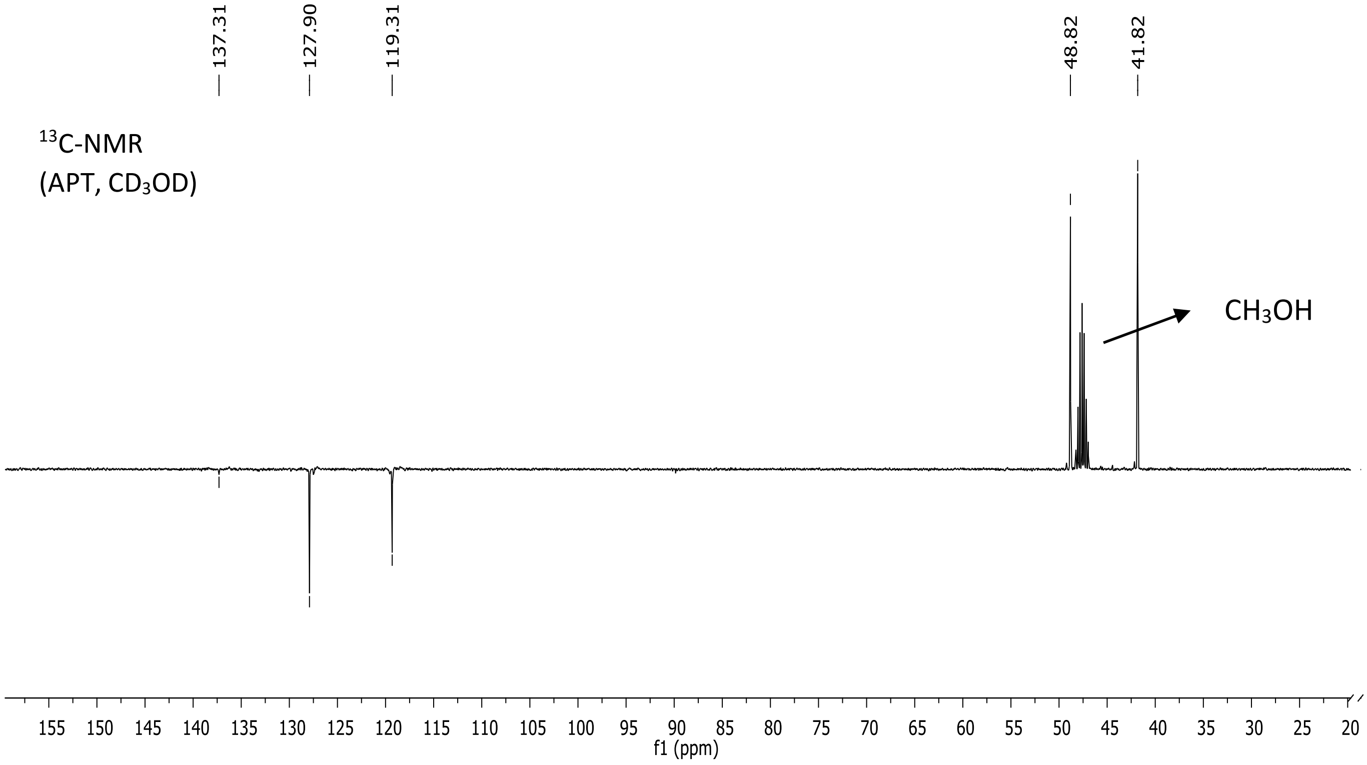

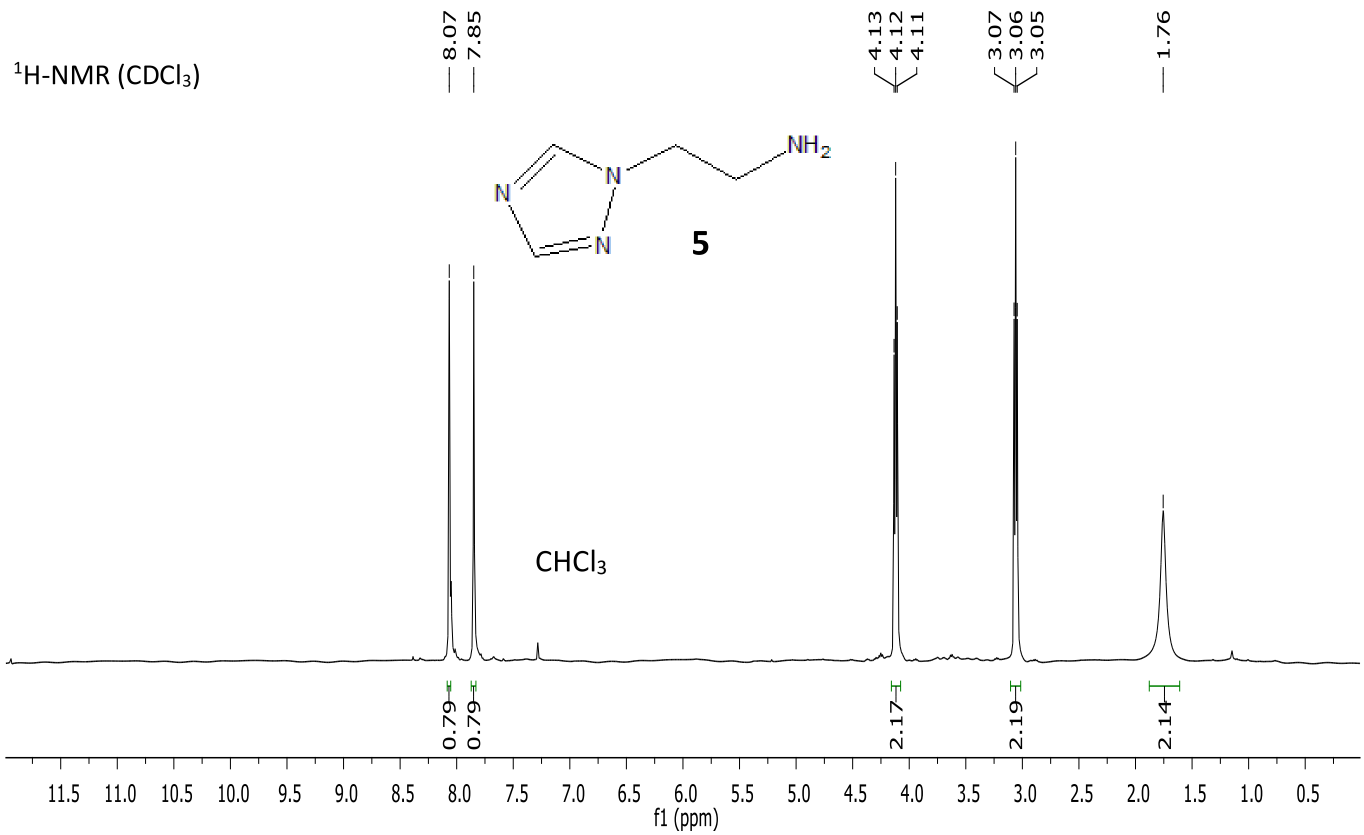

^{1}H-NMR ($CDCl_3$)
8.07
7.85
4.13
4.12
4.11
3.07
3.06
3.05
1.76
NH_2
N
N
N
5
$CHCl_3$
0.79
0.79
2.17
2.19
2.14
11.5 11.0 10.5 10.0 9.5 9.0 8.5 8.0 7.5 7.0 6.5 6.0 5.5 5.0 4.5 4.0 3.5 3.0 2.5 2.0 1.5 1.0 0.5
f1 (ppm)

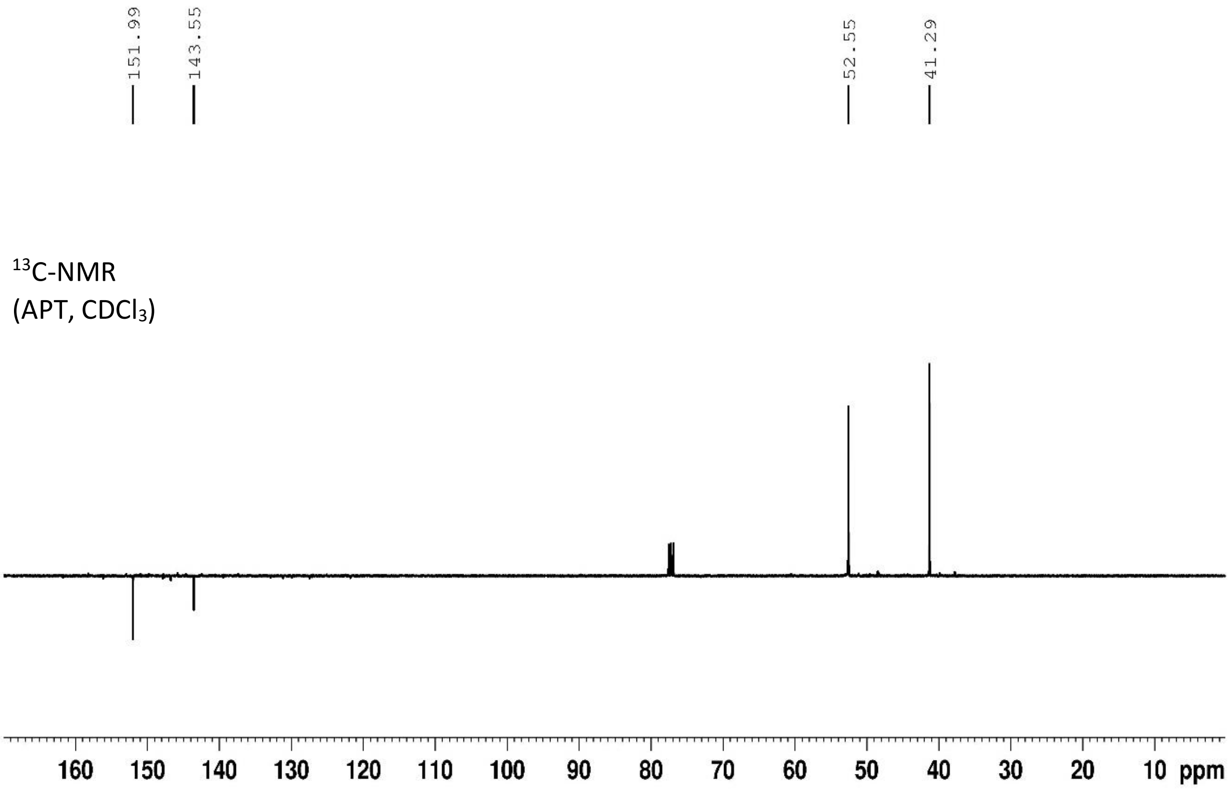

151.99
143.55
52.55
41.29
^{13}C-NMR
(APT, $CDCl_3$)
160 150 140 130 120 110 100 90 80 70 60 50 40 30 20 10 ppm

^{1}H-NMR (D_2O)

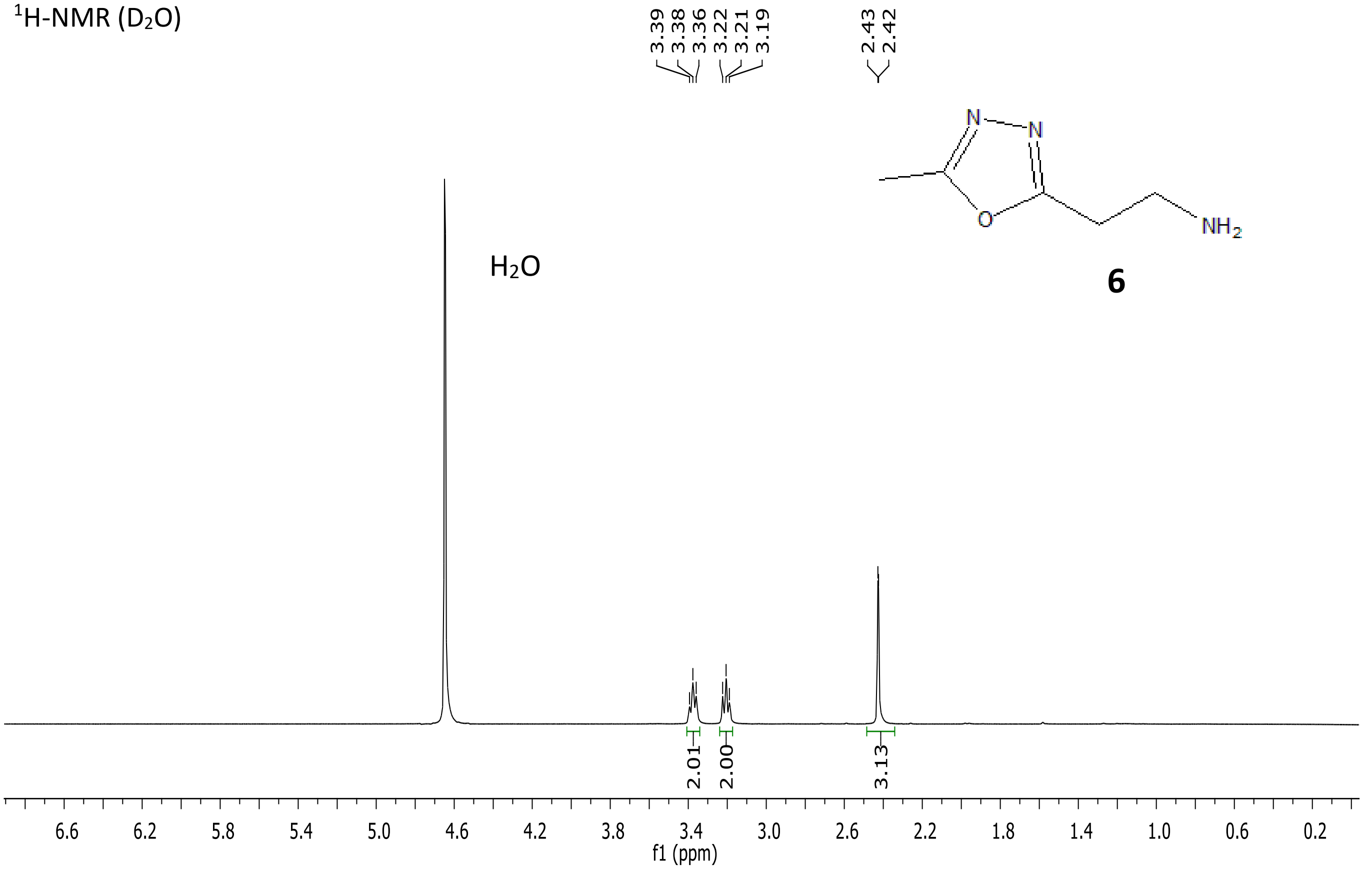

6

6 (oxalate salt)

^{13}C-NMR

(Proton decoupled, D_2O)

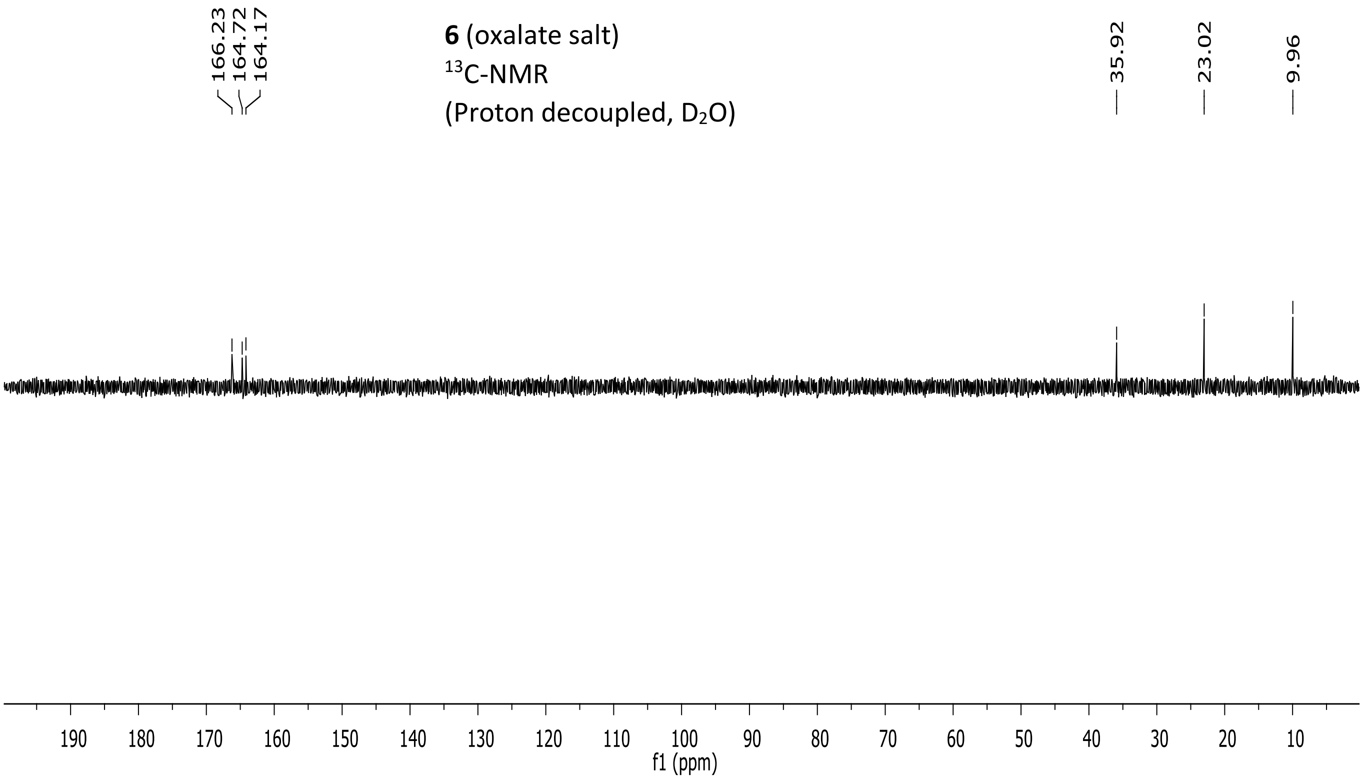

^{1}H-NMR ($CDCl_3$)

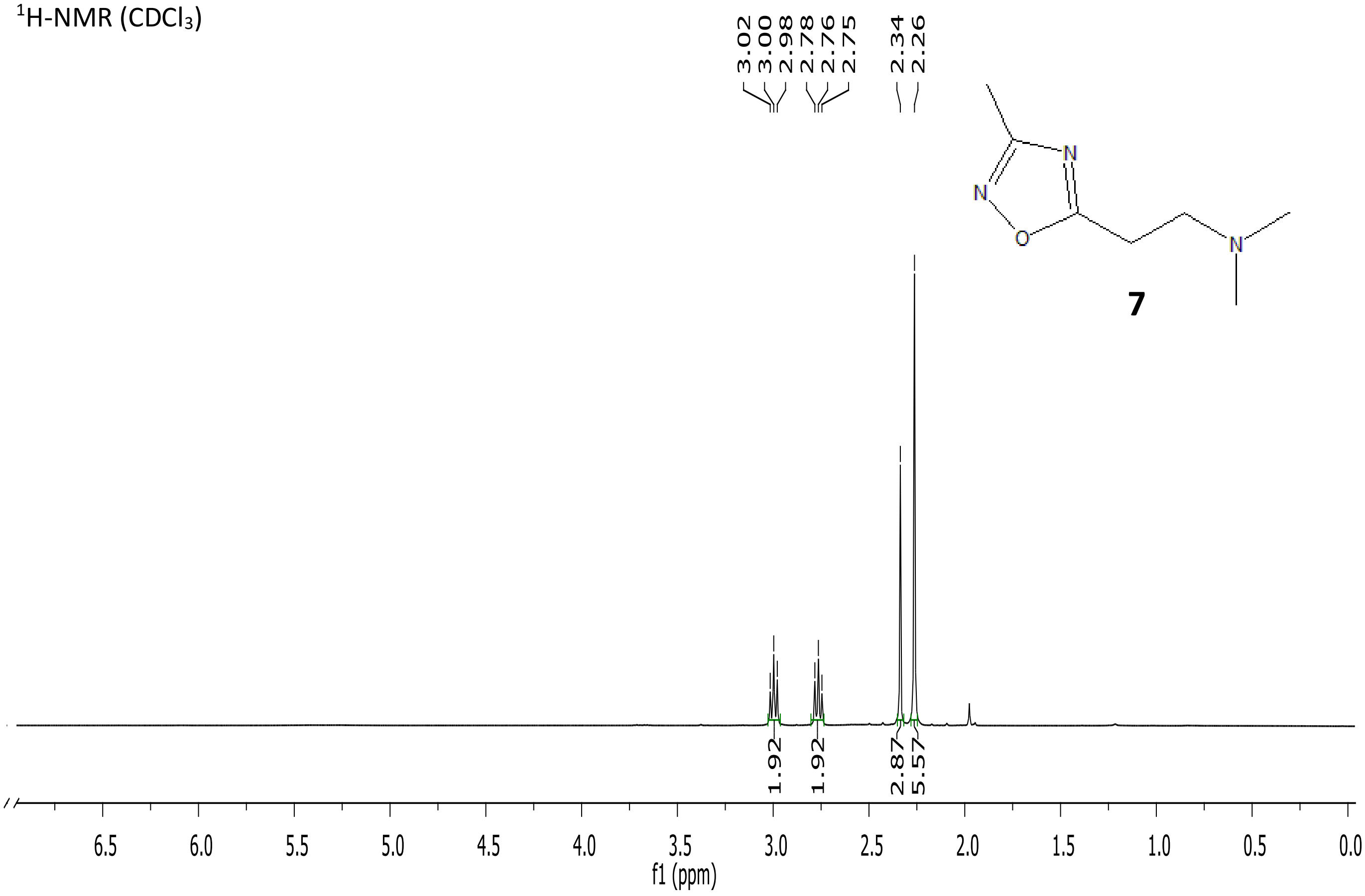

7

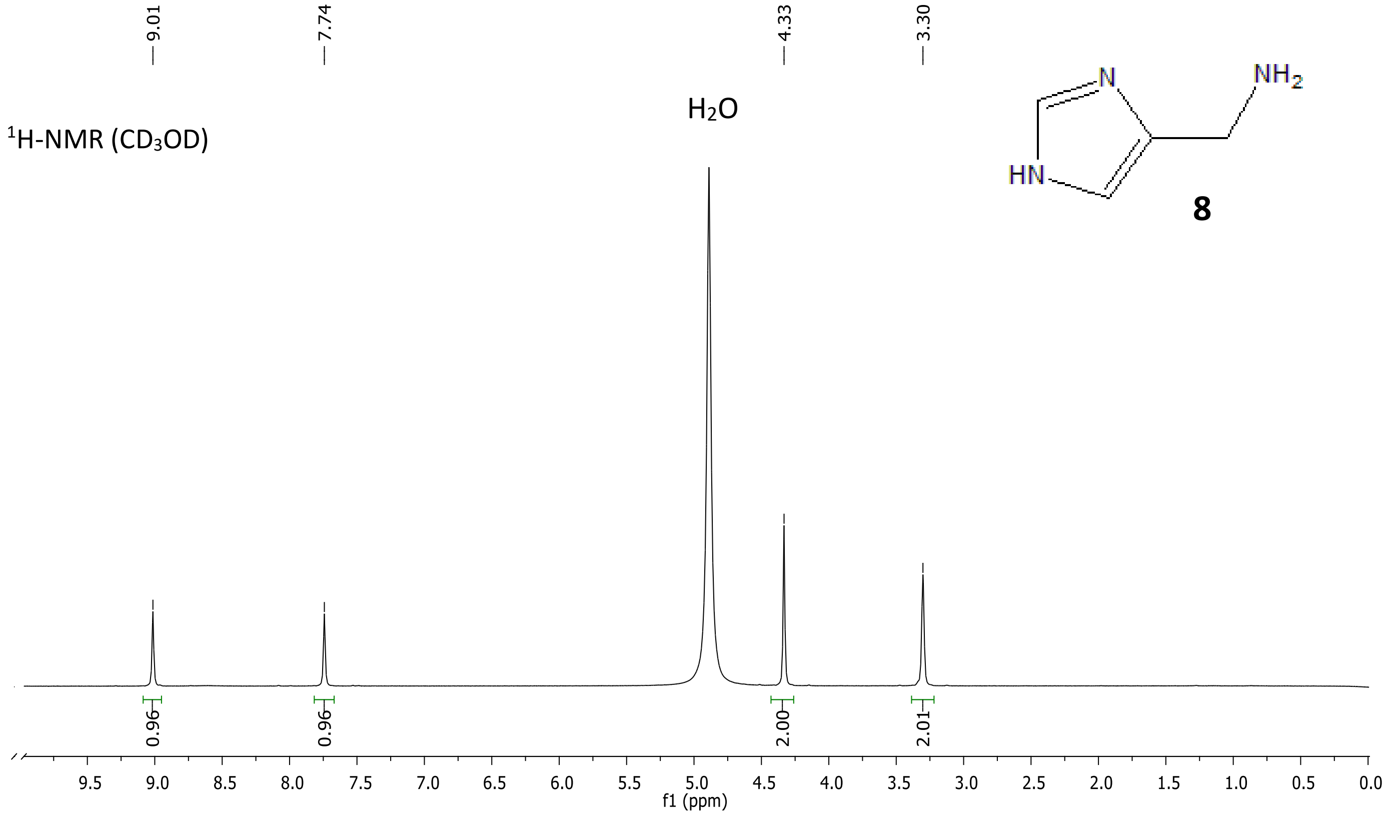

9.01
7.74
4.33
3.30
H_2O
^{1}H-NMR (CD_3OD)
N
NH_2
HN
8
0.96
0.96
2.00
2.01
9.5 9.0 8.5 8.0 7.5 7.0 6.5 6.0 5.5 5.0 4.5 4.0 3.5 3.0 2.5 2.0 1.5 1.0 0.5 0.0
f1 (ppm)

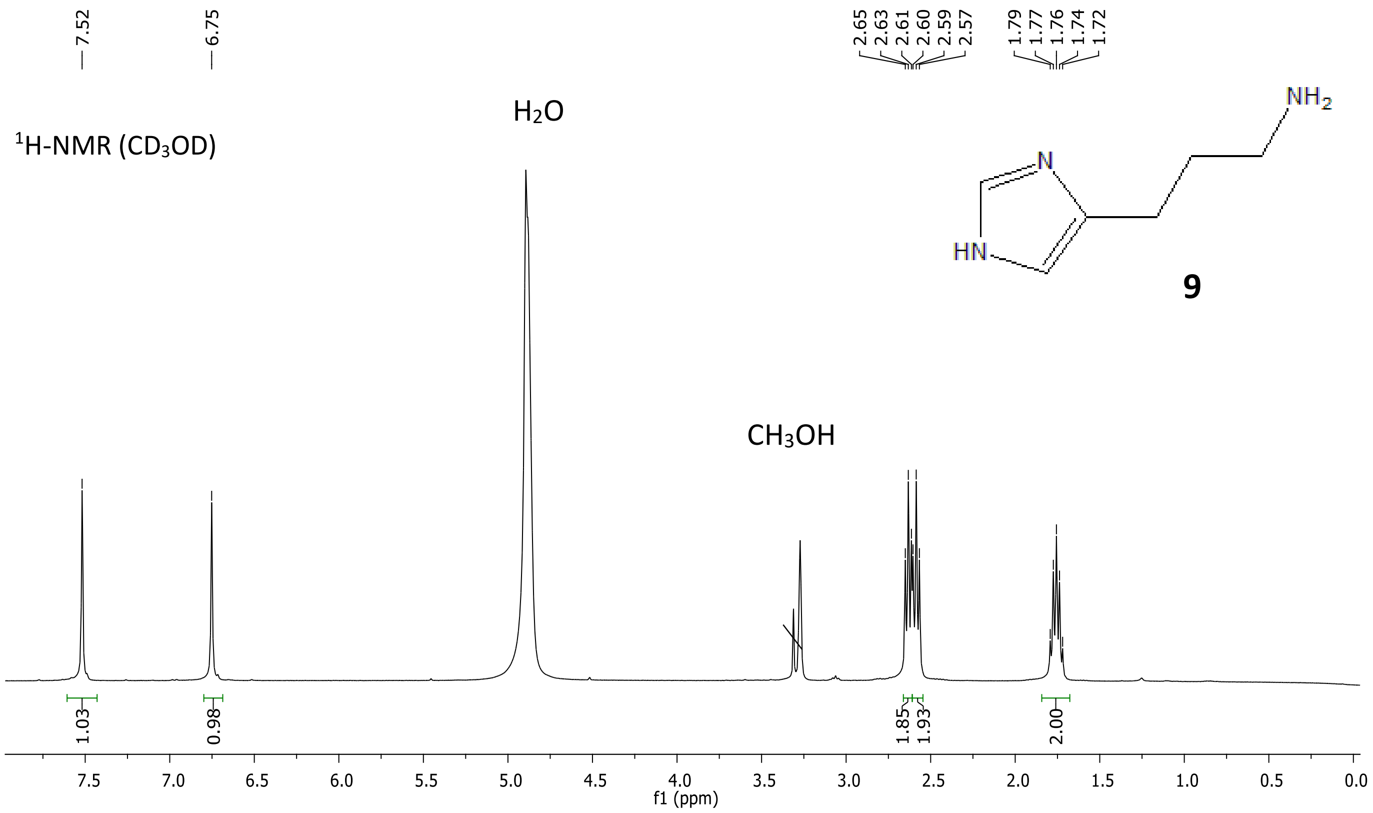

1H-NMR (CD3OD)
H2O
CH3OH
7.52
6.75
2.65 2.63 2.61 2.60 2.59 2.57
1.79 1.77 1.76 1.74 1.72
1.03
0.98
1.85
1.93
2.00
7.5 7.0 6.5 6.0 5.5 5.0 4.5 4.0 3.5 3.0 2.5 2.0 1.5 1.0 0.5 0.0
f1 (ppm)
NH2
N
HN
9

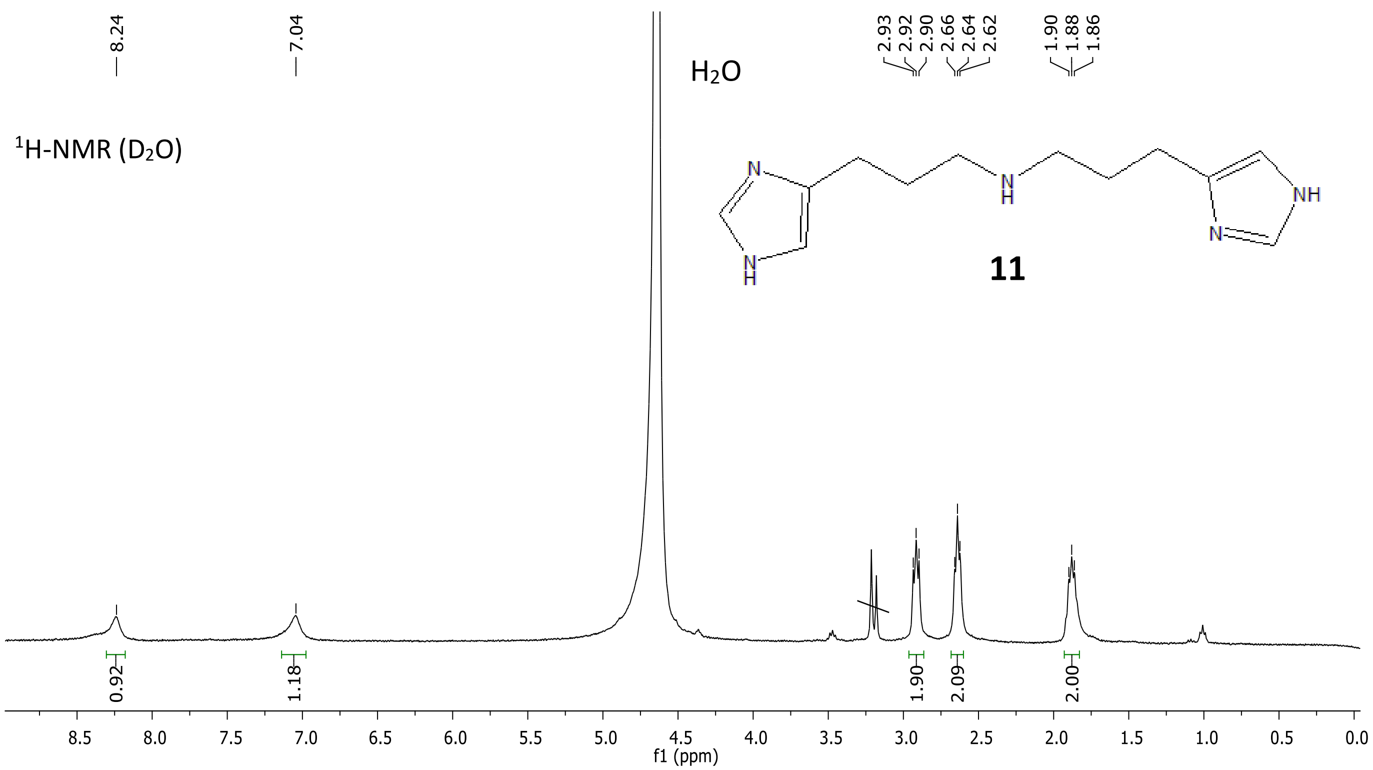

8.24
7.04
H2O
2.93
2.92
2.90
2.66
2.64
2.62
1.90
1.88
1.86
1H-NMR (D2O)
N
NH
11
0.92
1.18
1.90
2.09
2.00
f1 (ppm)

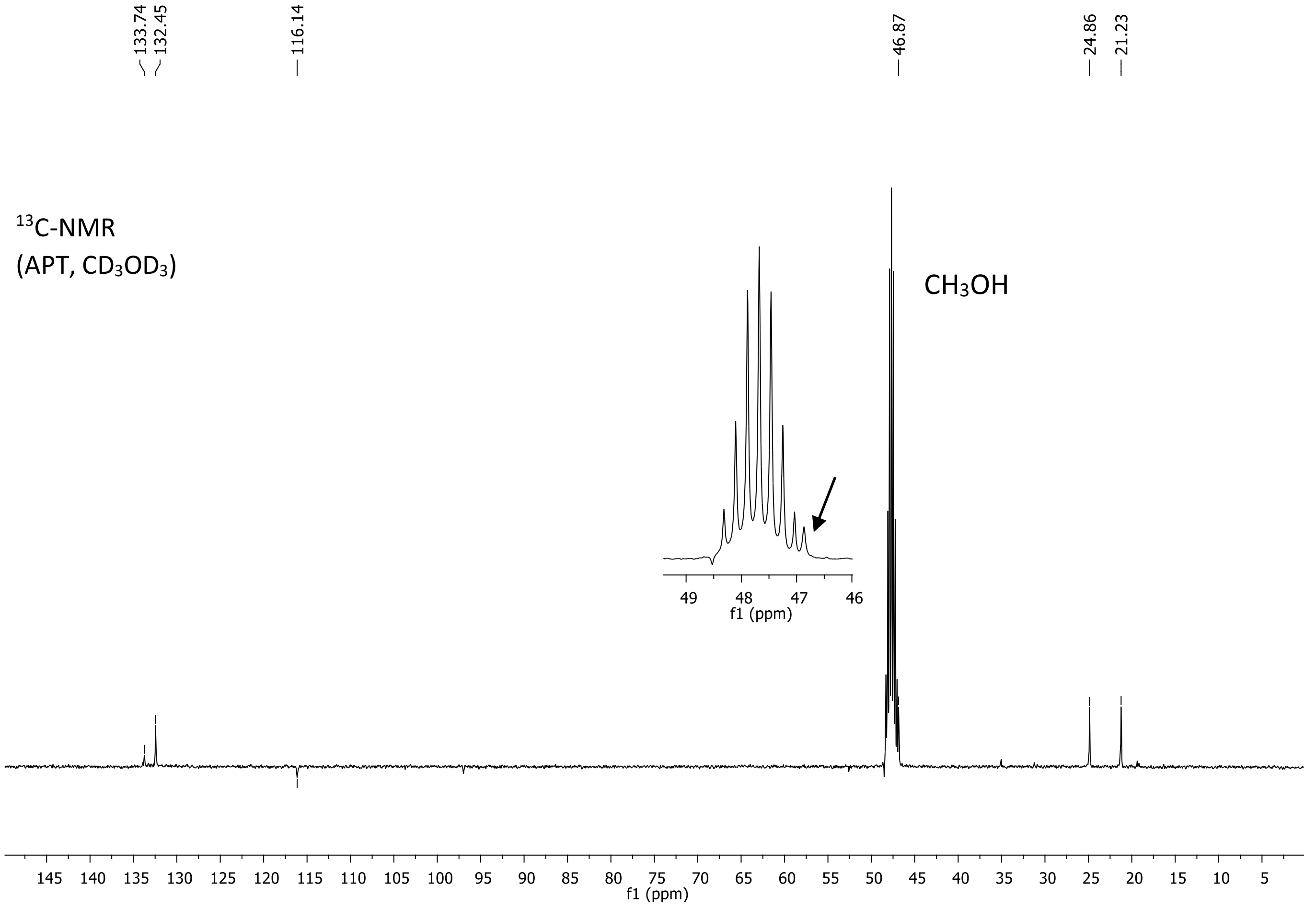

133.74
132.45
116.14
46.87
24.86
21.23
13C-NMR
(APT, CD3OD3)
CH3OH
f1 (ppm)

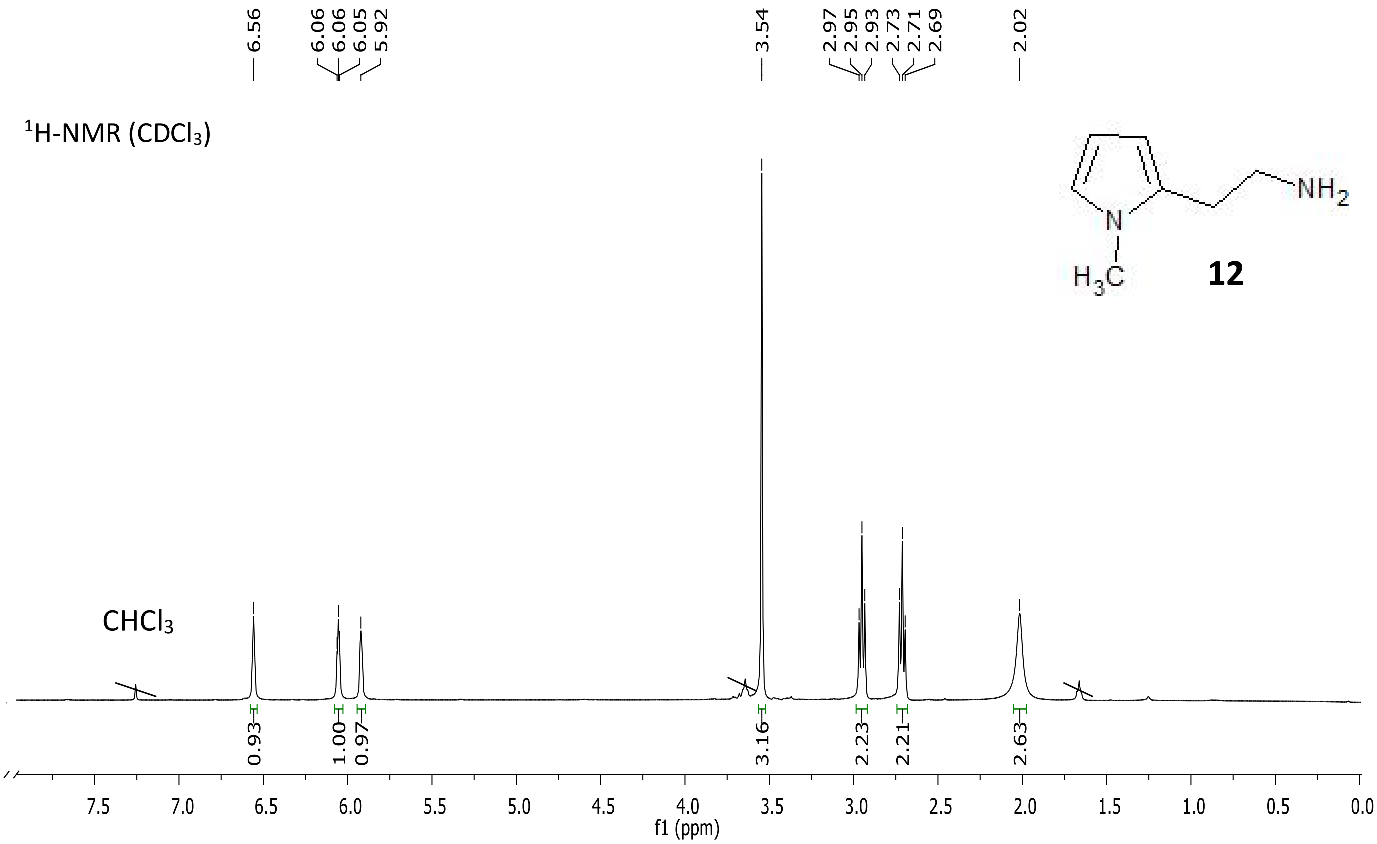

^{1}H-NMR (CDCl$_3$)
6.56
6.06
6.06
6.05
5.92
3.54
2.97
2.95
2.93
2.73
2.71
2.69
2.02
N
NH$_2$
H$_3$C
CHCl$_3$
0.93
1.00
0.97
3.16
2.23
2.21
2.63
7.5 7.0 6.5 6.0 5.5 5.0 4.5 4.0 3.5 3.0 2.5 2.0 1.5 1.0 0.5 0.0
f1 (ppm)

12

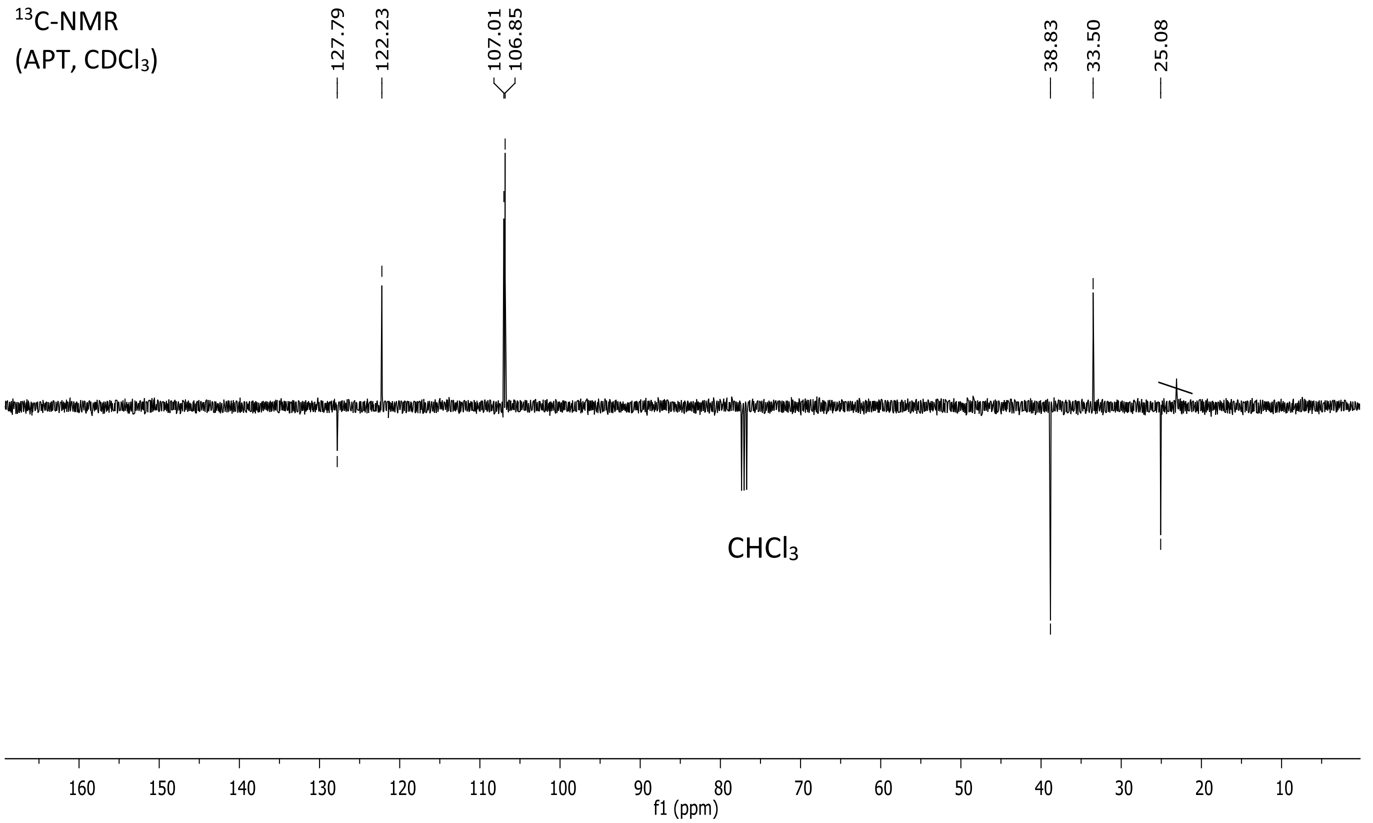

^{13}C-NMR
(APT, CDCl$_3$)
127.79
122.23
107.01
106.85
38.83
33.50
25.08
CHCl$_3$
160 150 140 130 120 110 100 90 80 70 60 50 40 30 20 10
f1 (ppm)

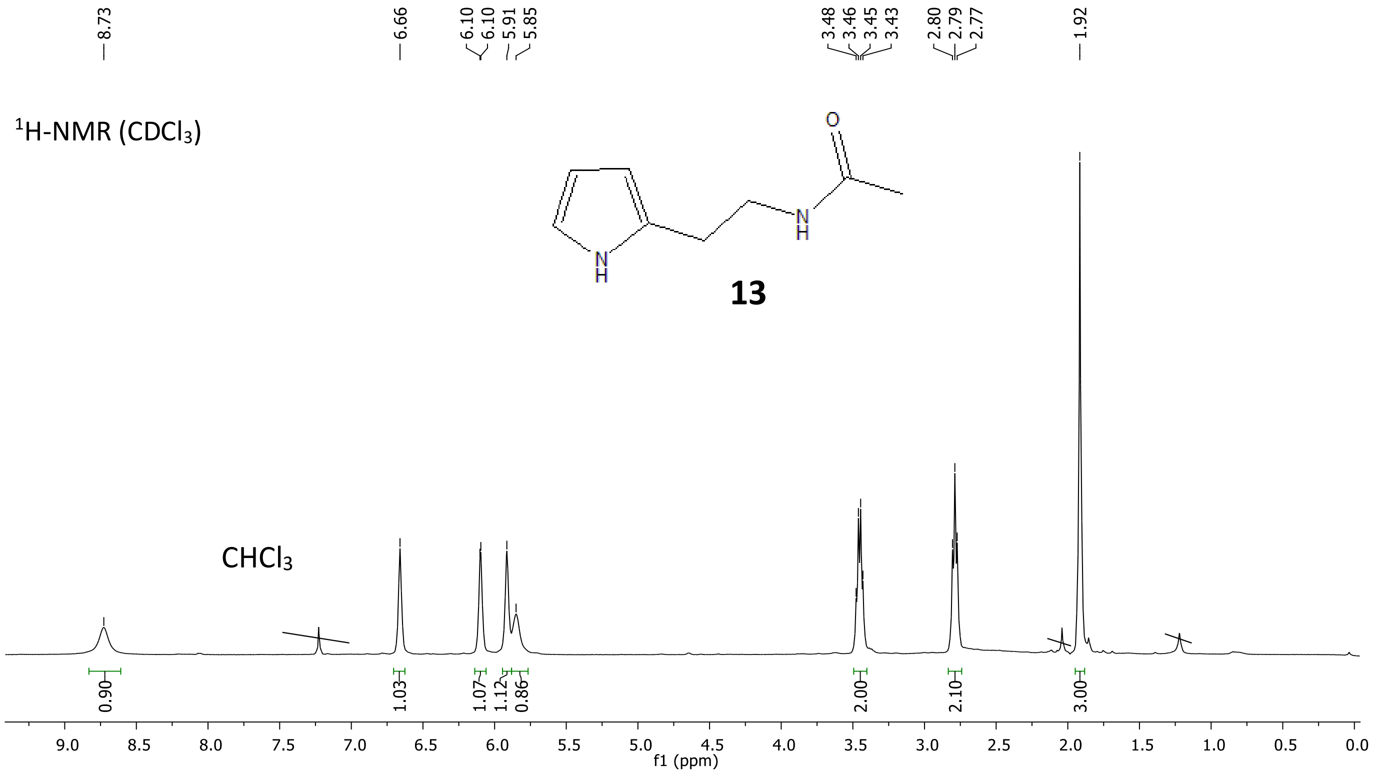

8.73
6.66
6.10
6.10
5.91
5.85
3.48
3.46
3.45
3.43
2.80
2.79
2.77
1.92
^{1}H-NMR ($CDCl_3$)
13
$CHCl_3$
0.90
1.03
1.07
1.12
0.86
2.00
2.10
3.00
9.0 8.5 8.0 7.5 7.0 6.5 6.0 5.5 5.0 4.5 4.0 3.5 3.0 2.5 2.0 1.5 1.0 0.5 0.0
f1 (ppm)

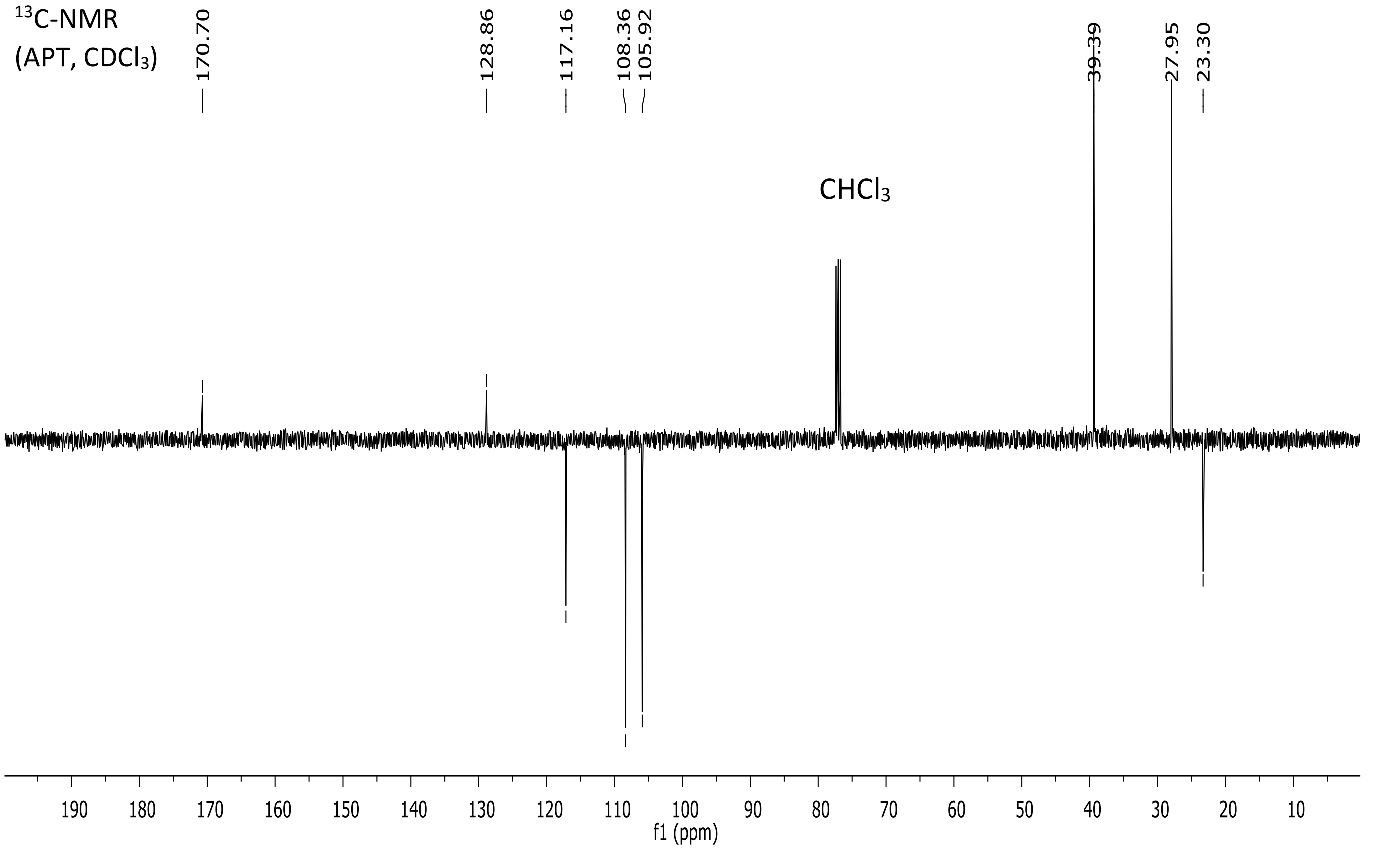

^{13}C-NMR
(APT, $CDCl_3$)
170.70
128.86
117.16
108.36
105.92
39.39
27.95
23.30
$CHCl_3$
190 180 170 160 150 140 130 120 110 100 90 80 70 60 50 40 30 20 10
f1 (ppm)

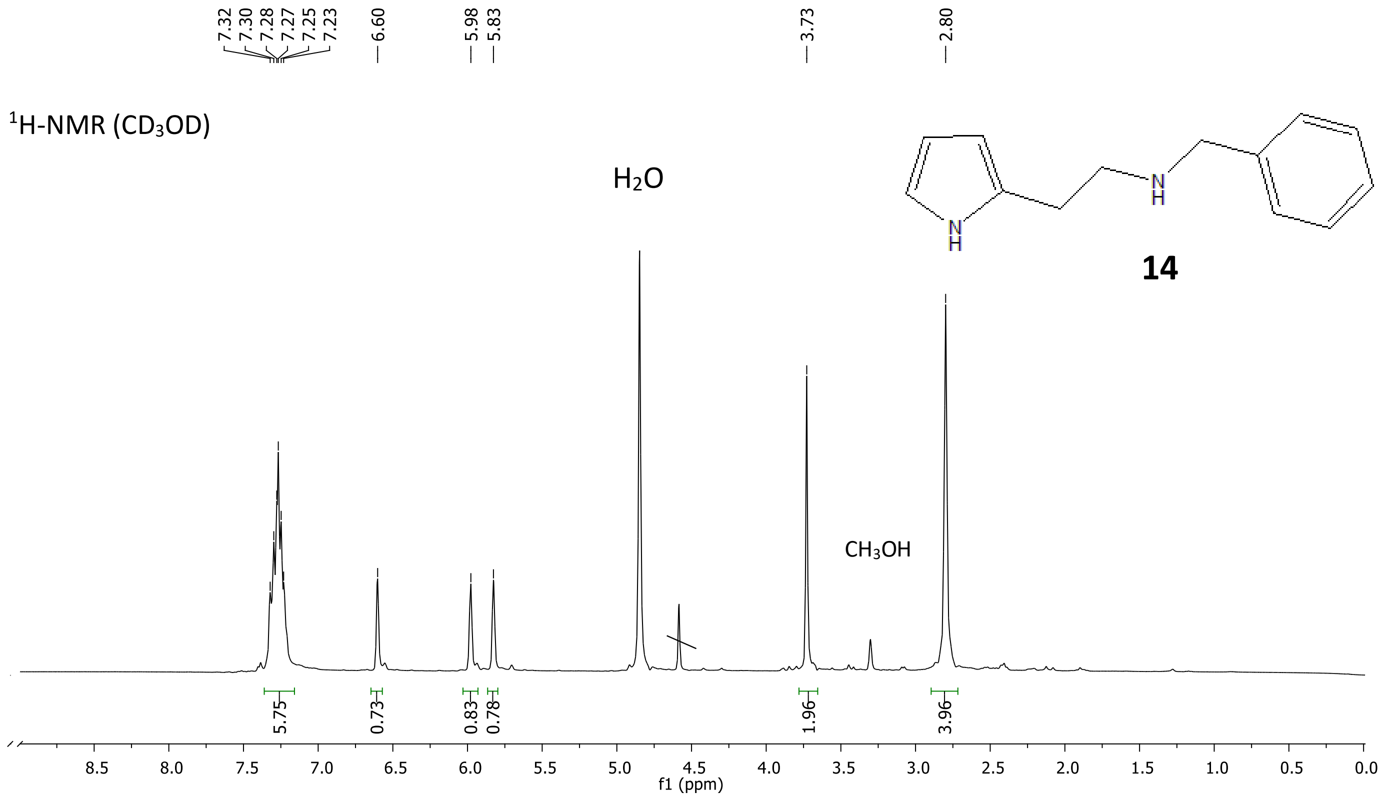

7.32
7.30
7.28
7.27
7.25
7.23
6.60
5.98
5.83
3.73
2.80
^{1}H-NMR (CD_3OD)
H_2O
14
CH_3OH
5.75
0.73
0.83
0.78
1.96
3.96
8.5 8.0 7.5 7.0 6.5 6.0 5.5 5.0 4.5 4.0 3.5 3.0 2.5 2.0 1.5 1.0 0.5 0.0
f1 (ppm)

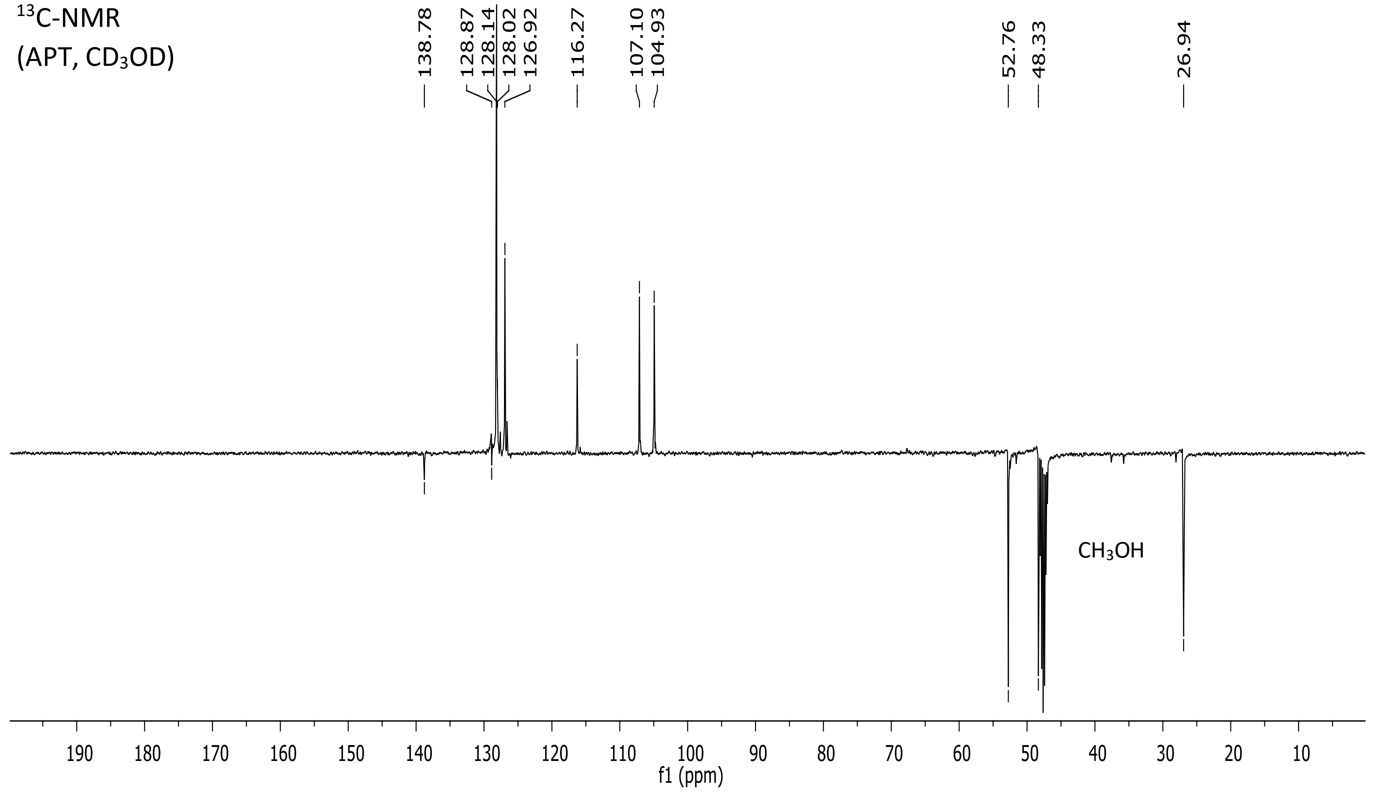

^{13}C-NMR
(APT, CD_3OD)
138.78
128.87
128.14
128.02
126.92
116.27
107.10
104.93
52.76
48.33
26.94
CH_3OH
190 180 170 160 150 140 130 120 110 100 90 80 70 60 50 40 30 20 10
f1 (ppm)

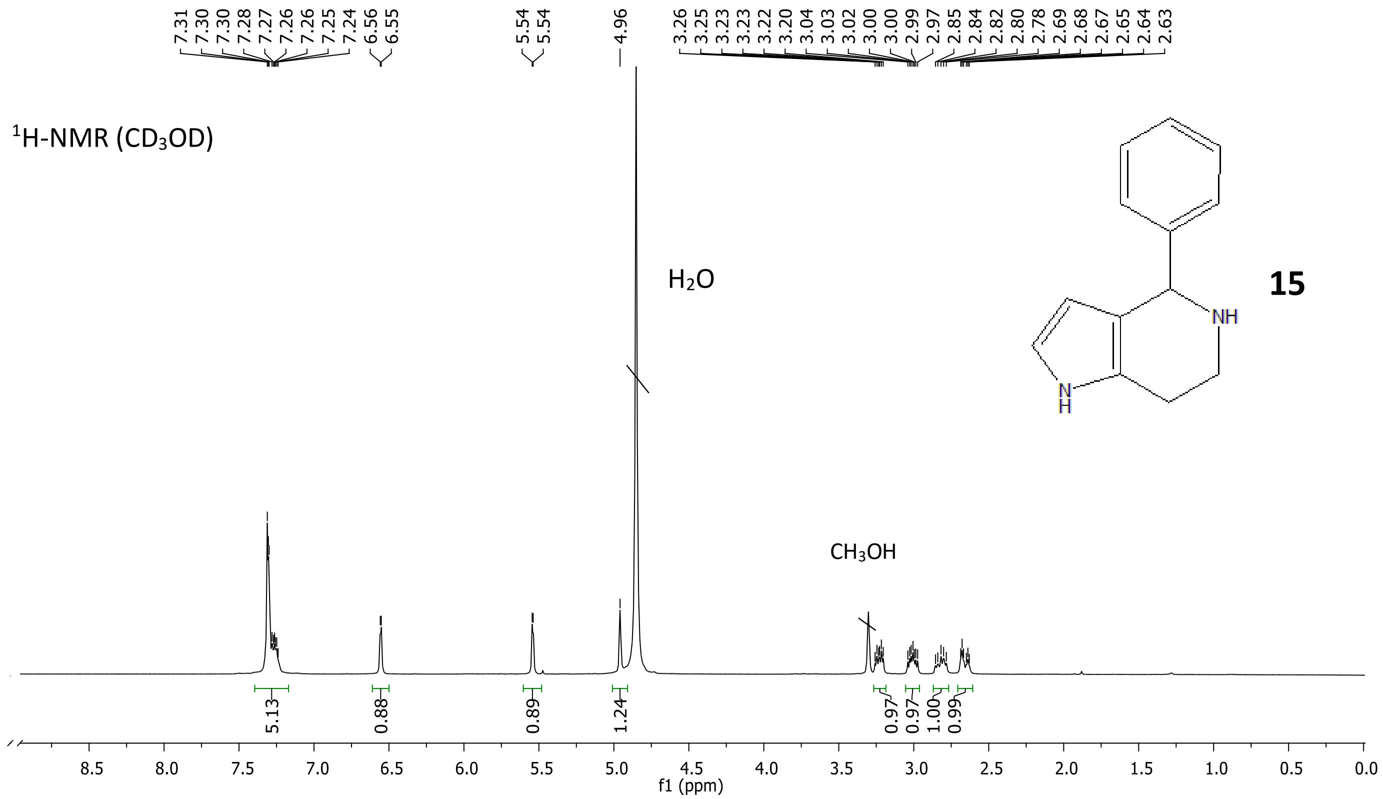

^{1}H-NMR (CD_3OD)
7.31 7.30 7.30 7.28 7.27 7.26 7.26 7.25 7.24 6.56 6.55
5.54 5.54
4.96
3.26 3.25 3.23 3.23 3.22 3.20 3.04 3.03 3.02 3.00 3.00 2.99 2.97 2.85 2.84 2.82 2.80 2.78 2.69 2.68 2.67 2.65 2.64 2.63
H_2O
15
NH
N
H
CH_3OH
5.13
0.88
0.89
1.24
0.97
0.97
1.00
0.99
8.5 8.0 7.5 7.0 6.5 6.0 5.5 5.0 4.5 4.0 3.5 3.0 2.5 2.0 1.5 1.0 0.5 0.0
f1 (ppm)

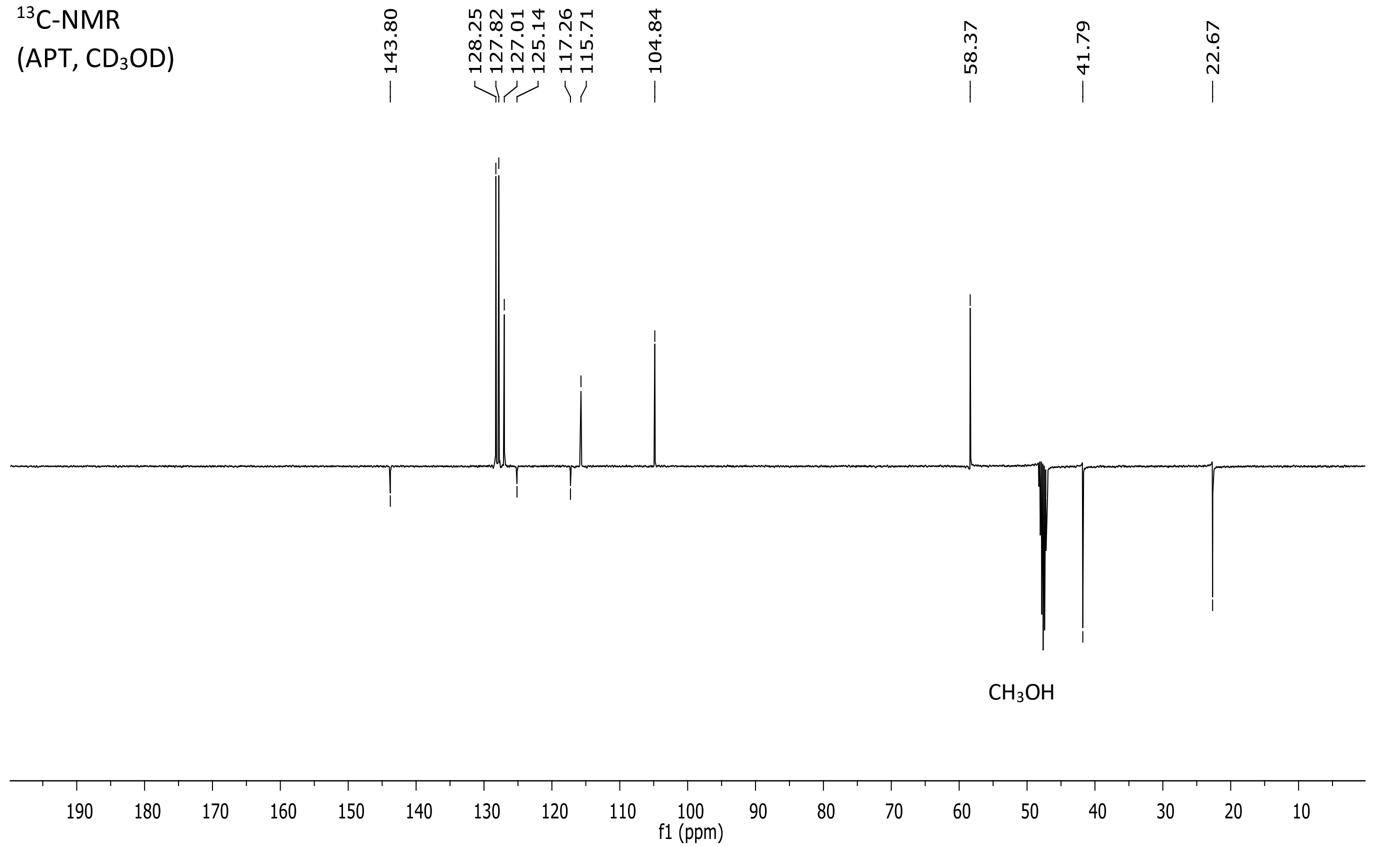

^{13}C-NMR
(APT, CD_3OD)
143.80
128.25 127.82 127.01 125.14 117.26 115.71
104.84
58.37
41.79
22.67
CH_3OH
190 180 170 160 150 140 130 120 110 100 90 80 70 60 50 40 30 20 10
f1 (ppm)

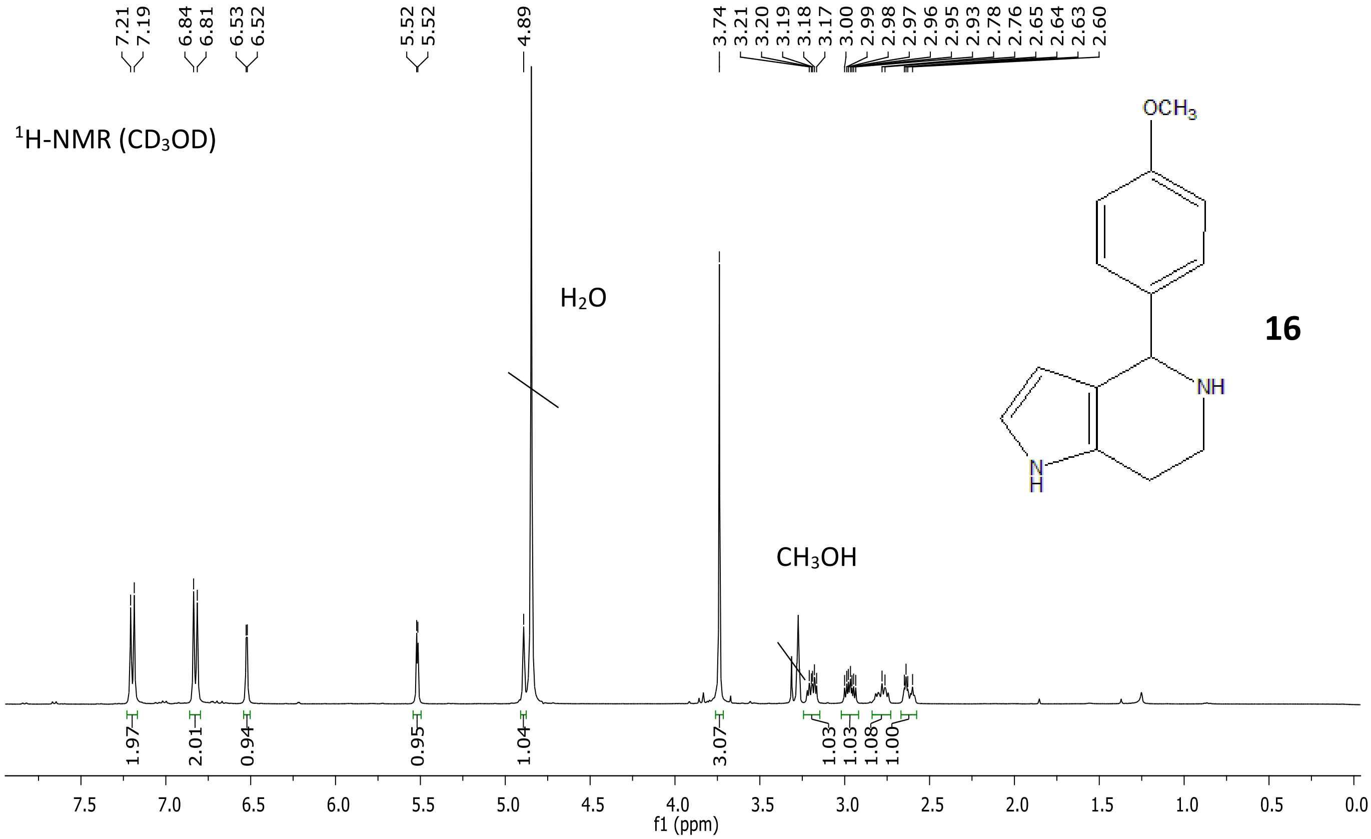

7.21
7.19
6.84
6.81
6.53
6.52
5.52
5.52
4.89
3.74
3.21
3.20
3.19
3.18
3.17
3.00
2.99
2.98
2.97
2.96
2.95
2.93
2.78
2.76
2.65
2.64
2.63
2.60
^{1}H-NMR (CD_3OD)
H_2O
CH_3OH
OCH3
16
NH
N
H
1.97
2.01
0.94
0.95
1.04
3.07
1.03
1.03
1.08
1.00
f1 (ppm)

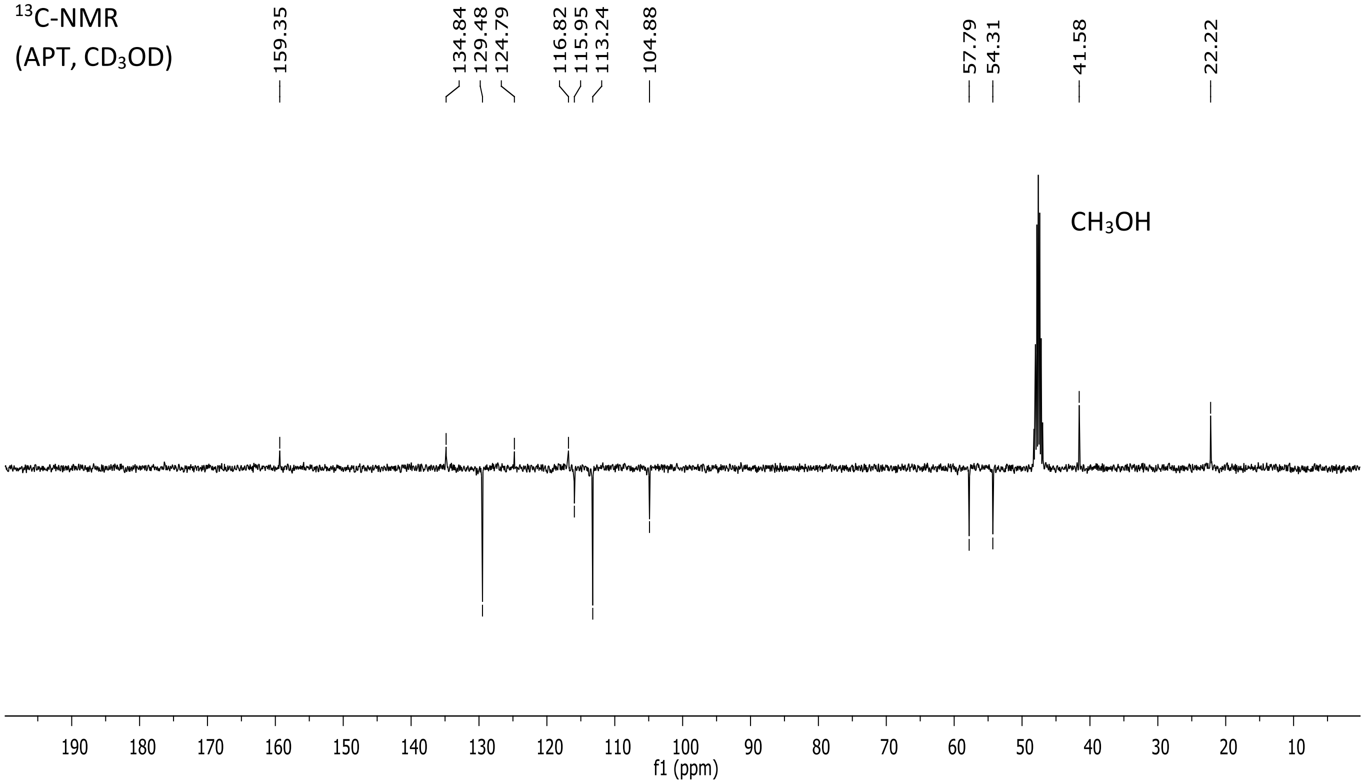

^{13}C-NMR
(APT, CD_3OD)
159.35
134.84
129.48
124.79
116.82
115.95
113.24
104.88
57.79
54.31
41.58
22.22
CH_3OH
f1 (ppm)

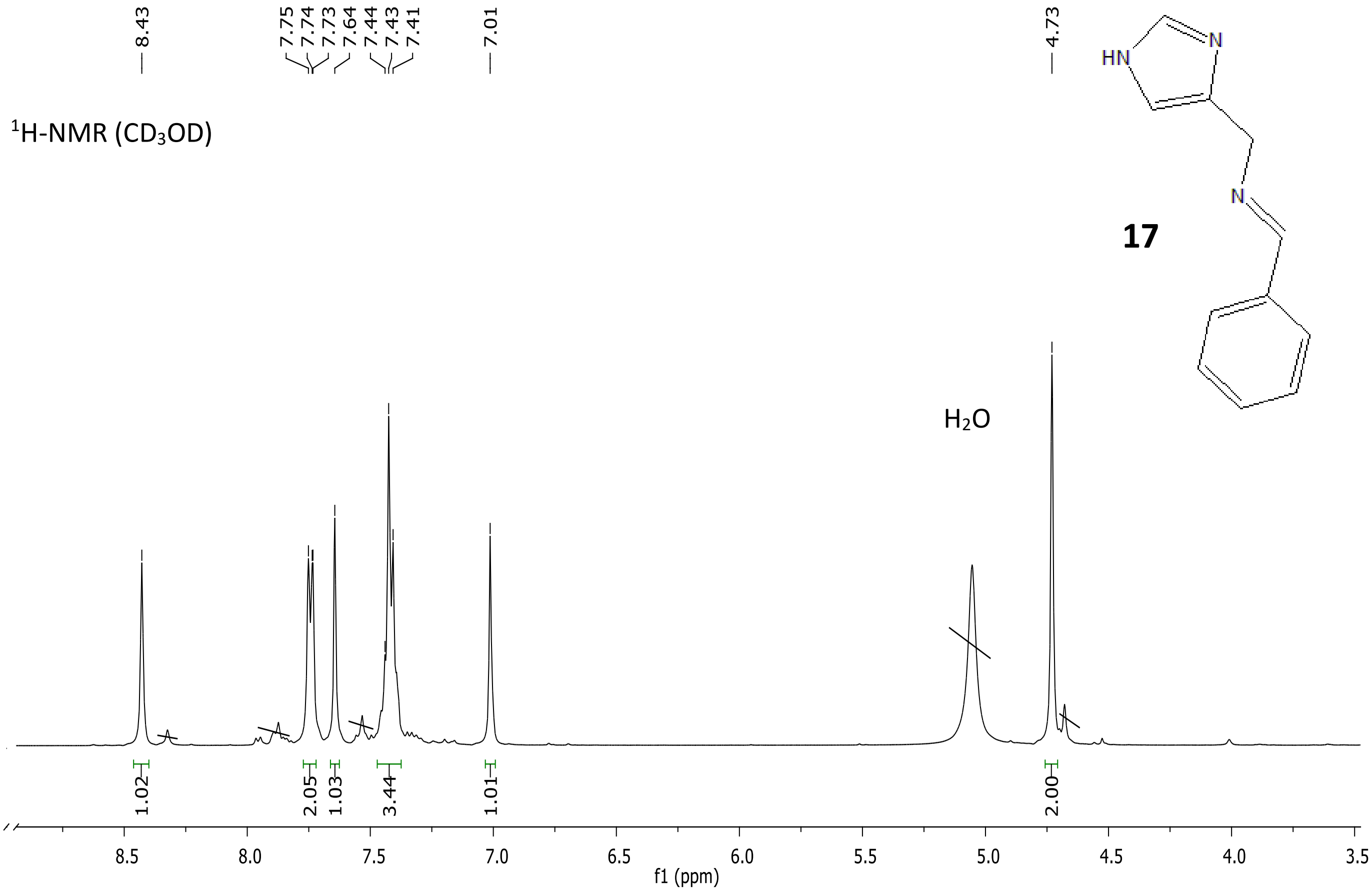

8.43
7.75
7.74
7.73
7.64
7.44
7.43
7.41
7.01
4.73
^1H-NMR (CD_3OD)
H_2O
1.02
2.05
1.03
3.44
1.01
2.00
8.5 8.0 7.5 7.0 6.5 6.0 5.5 5.0 4.5 4.0 3.5
f1 (ppm)
HN
N
N

17

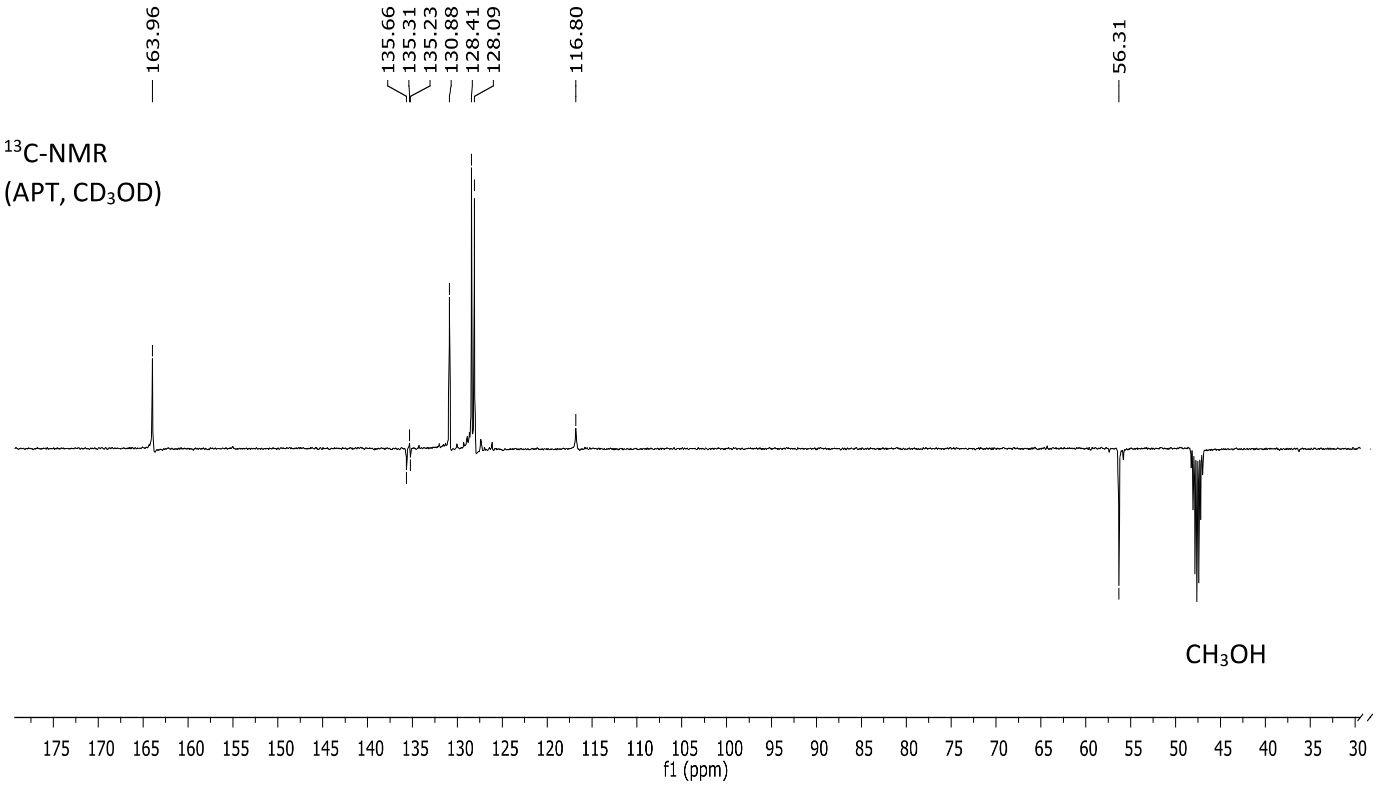

163.96
135.66
135.31
135.23
130.88
128.41
128.09
116.80
56.31
^{13}C-NMR
(APT, CD_3OD)
CH_3OH
175 170 165 160 155 150 145 140 135 130 125 120 115 110 105 100 95 90 85 80 75 70 65 60 55 50 45 40 35 30
f1 (ppm)

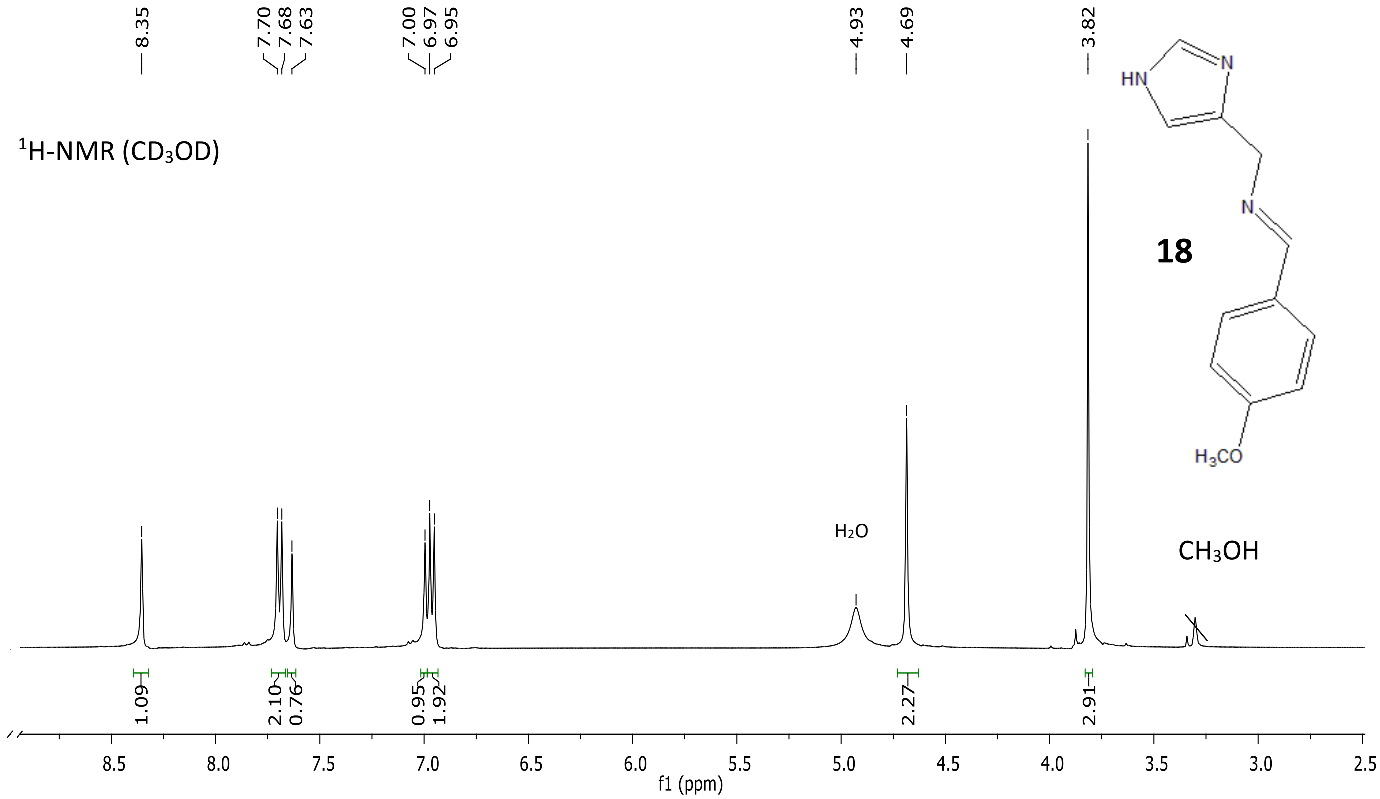
8.35
7.70
7.68
7.63
7.00
6.97
6.95
4.93
4.69
3.82
1H-NMR (CD3OD)
HN
N
N
18
H3CO
H2O
CH3OH
1.09
2.10
0.76
0.95
1.92
2.27
2.91
f1 (ppm)

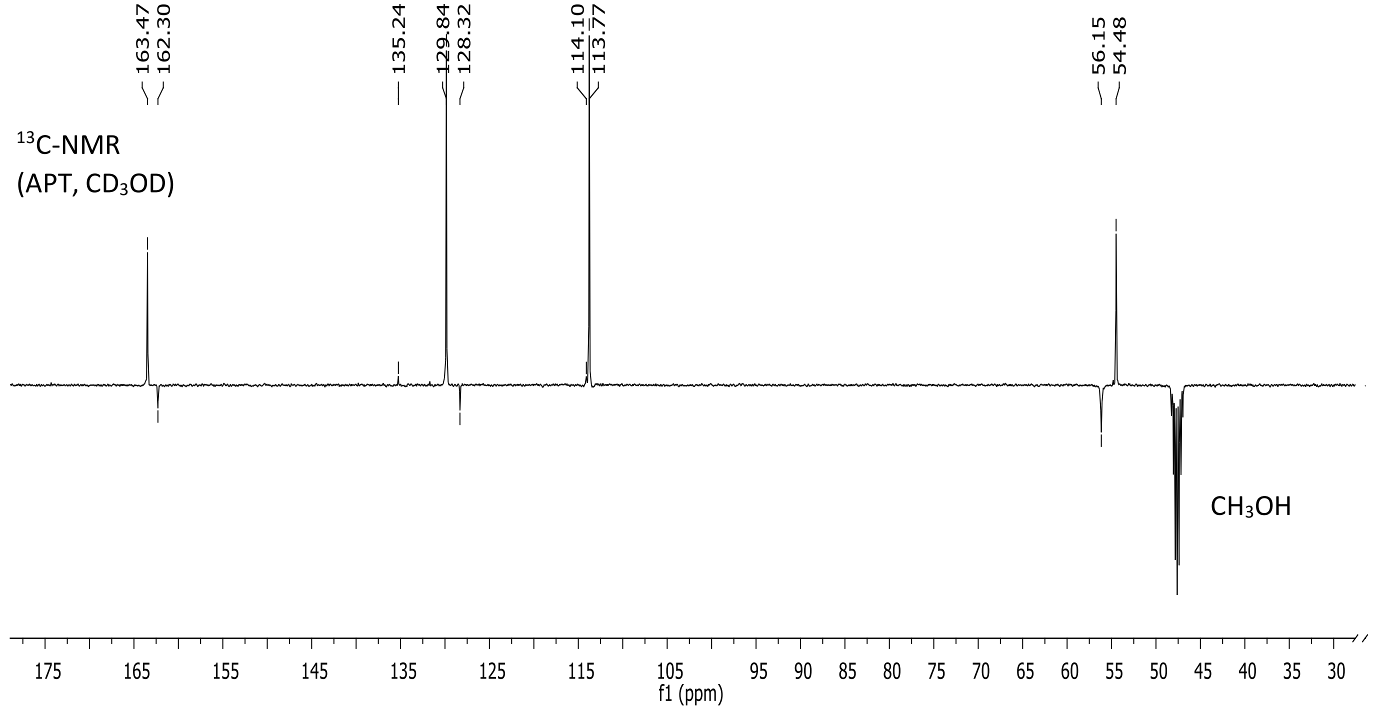
163.47
162.30
135.24
129.84
128.32
114.10
113.77
56.15
54.48
13C-NMR
(APT, CD3OD)
CH3OH
f1 (ppm)

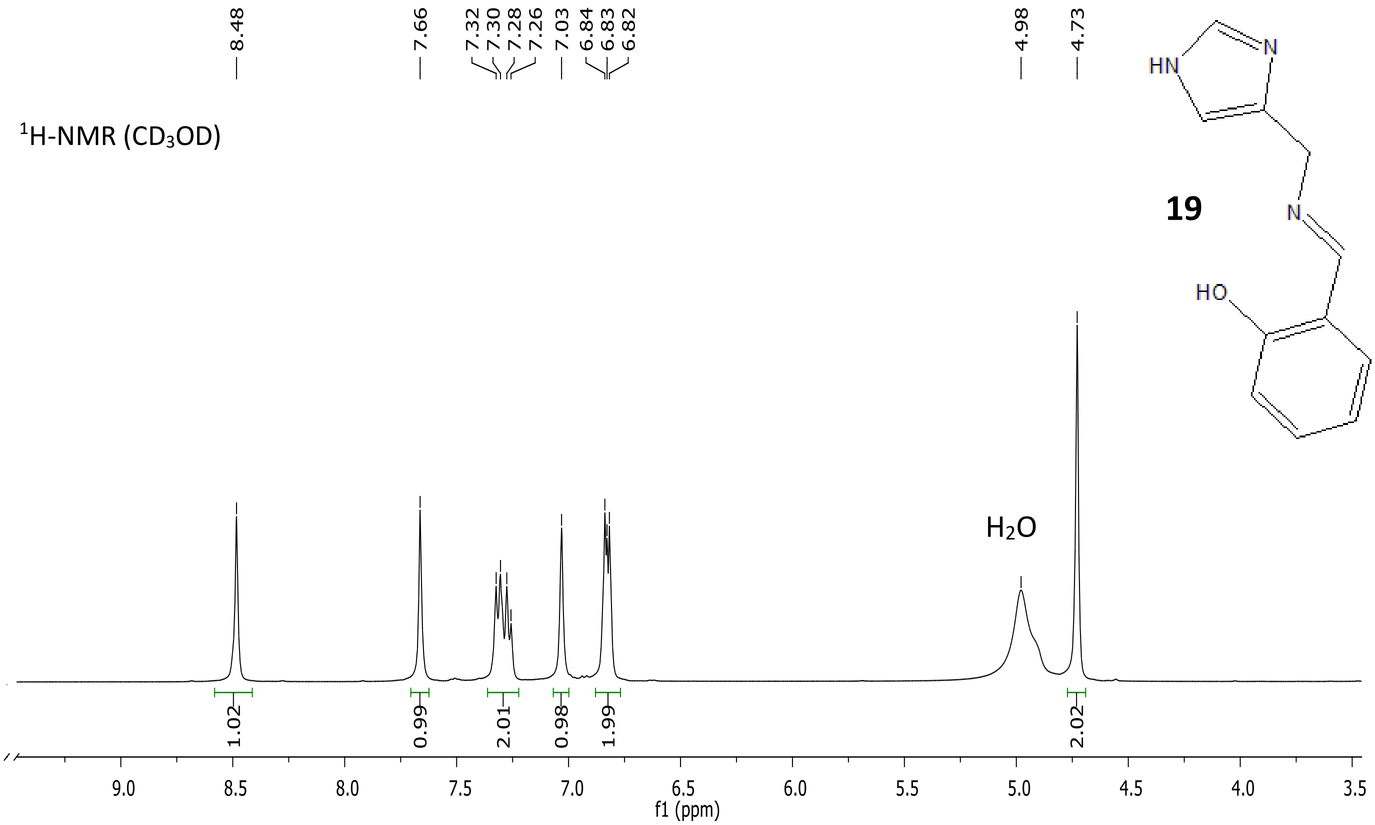

^1H-NMR (CD_3OD)
8.48
7.66
7.32
7.30
7.28
7.26
7.03
6.84
6.83
6.82
4.98
4.73
H_2O
1.02
0.99
2.01
0.98
1.99
2.02
9.0 8.5 8.0 7.5 7.0 6.5 6.0 5.5 5.0 4.5 4.0 3.5
f1 (ppm)
HN
N
N
HO
19

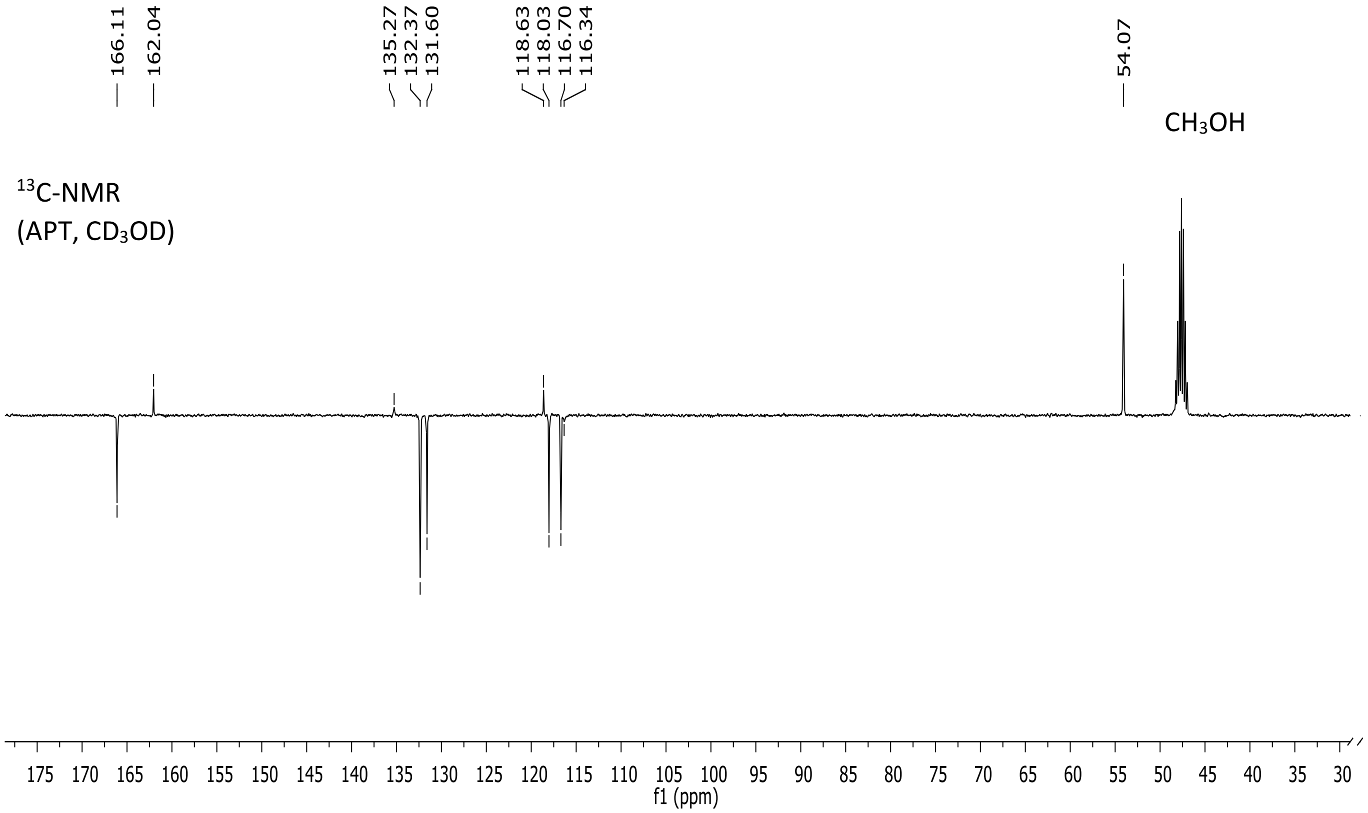

^{13}C-NMR
(APT, CD_3OD)
166.11
162.04
135.27
132.37
131.60
118.63
118.03
116.70
116.34
54.07
CH_3OH
175 170 165 160 155 150 145 140 135 130 125 120 115 110 105 100 95 90 85 80 75 70 65 60 55 50 45 40 35 30
f1 (ppm)

^{1}H-NMR (CD_3OD)

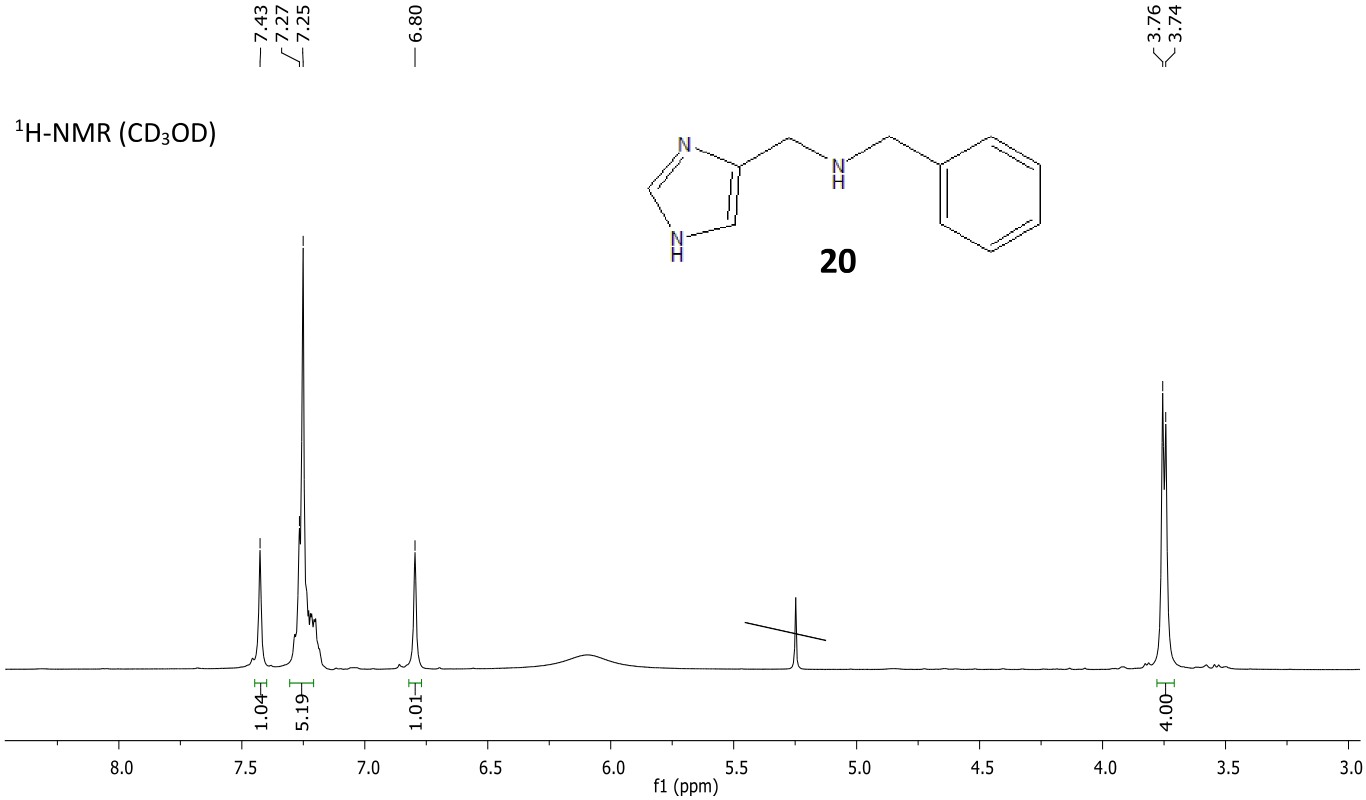

20

^{1}H-NMR (CD_3OD)

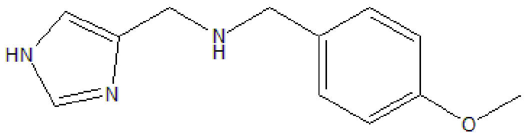

21

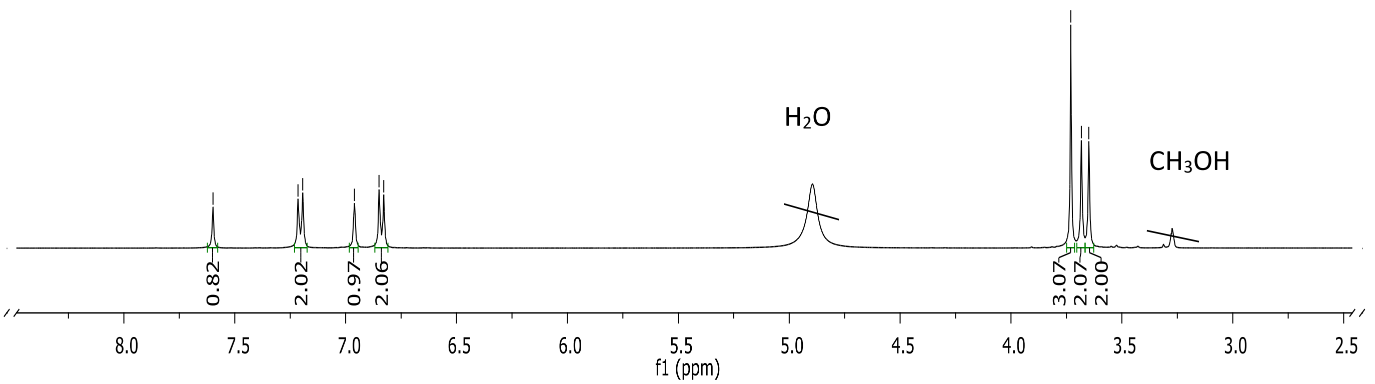

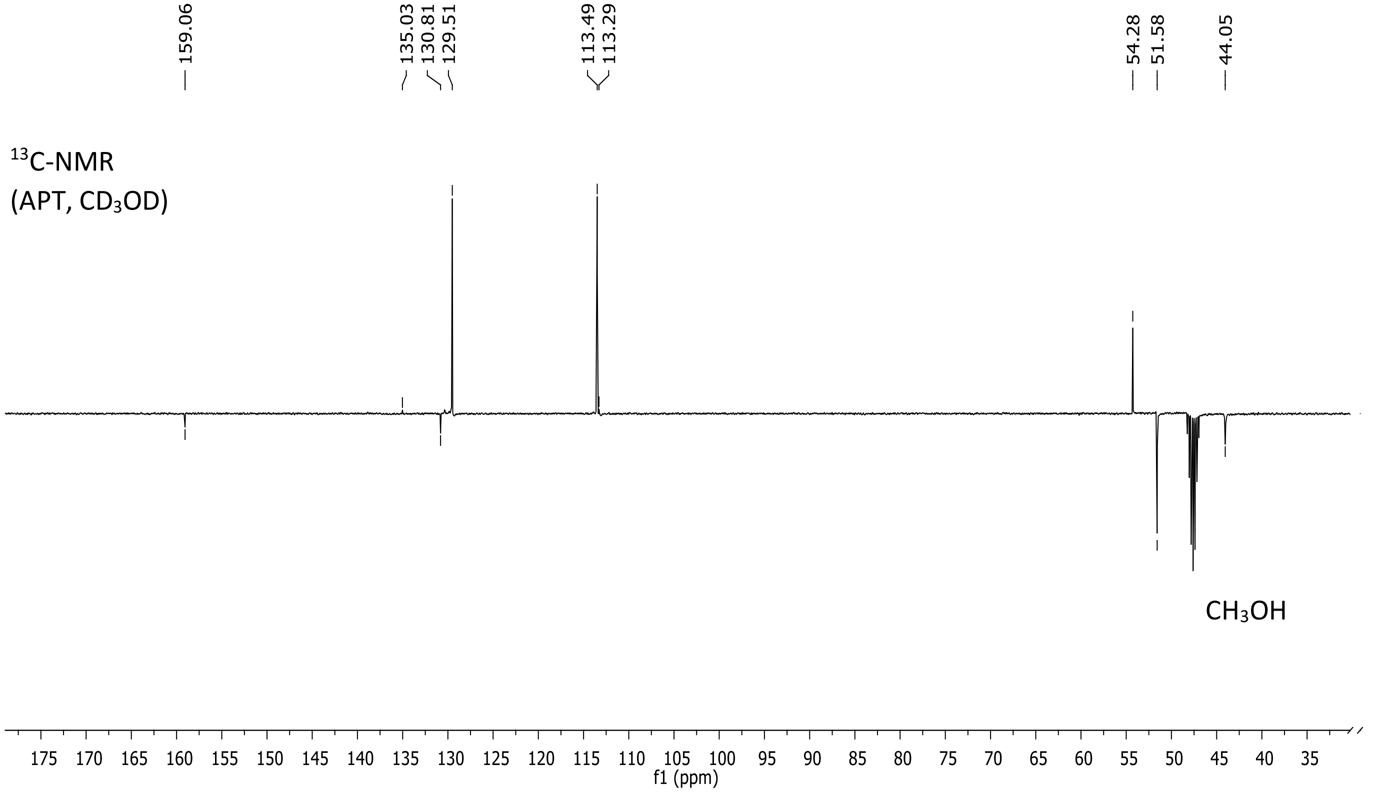

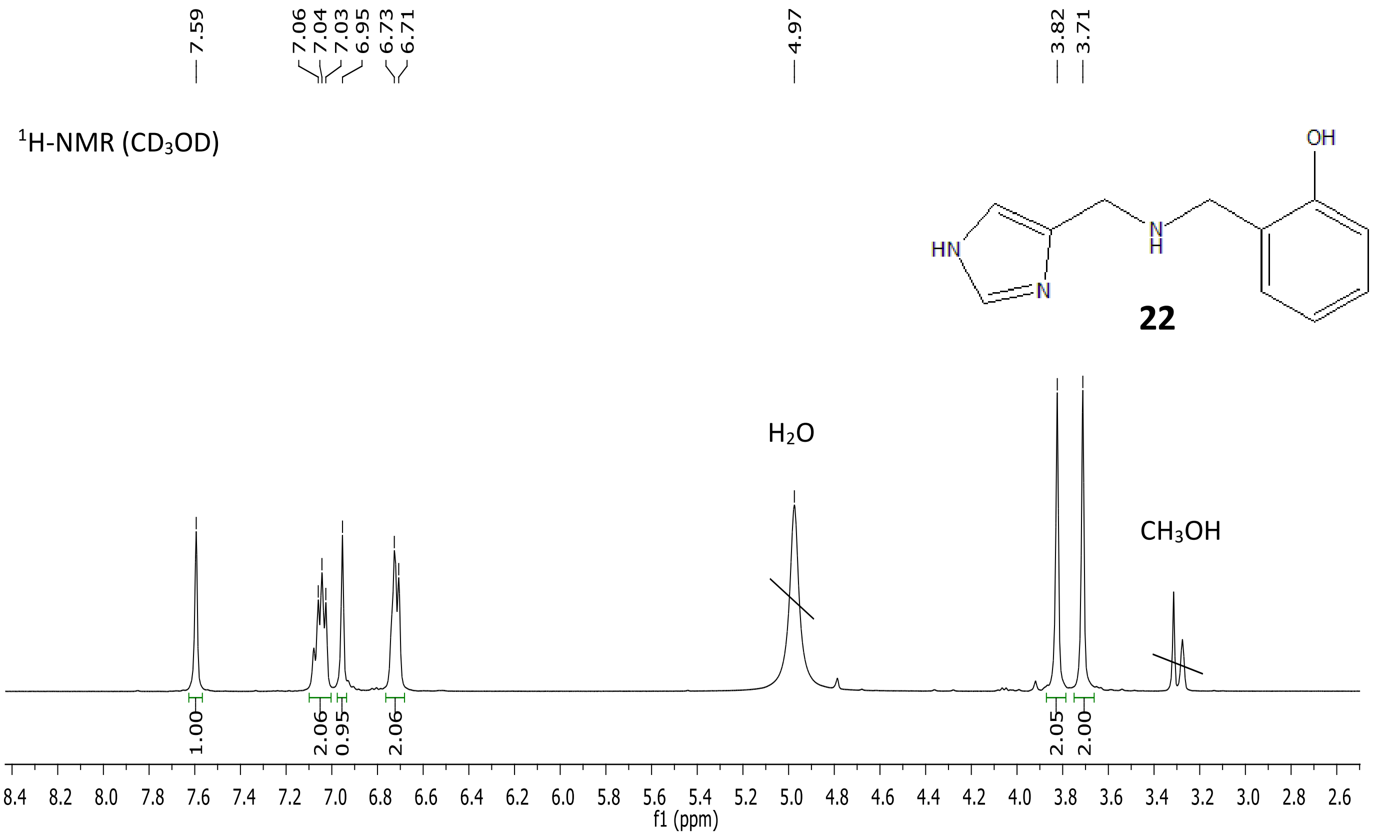
7.59
7.06
7.04
7.03
6.95
6.73
6.71
4.97
3.82
3.71
^{1}H-NMR (CD_3OD)
OH
HN
N
H
22
H_2O
CH_3OH
1.00
2.06
0.95
2.06
2.05
2.00
8.4 8.2 8.0 7.8 7.6 7.4 7.2 7.0 6.8 6.6 6.4 6.2 6.0 5.8 5.6 5.4 5.2 5.0 4.8 4.6 4.4 4.2 4.0 3.8 3.6 3.4 3.2 3.0 2.8 2.6
f1 (ppm)

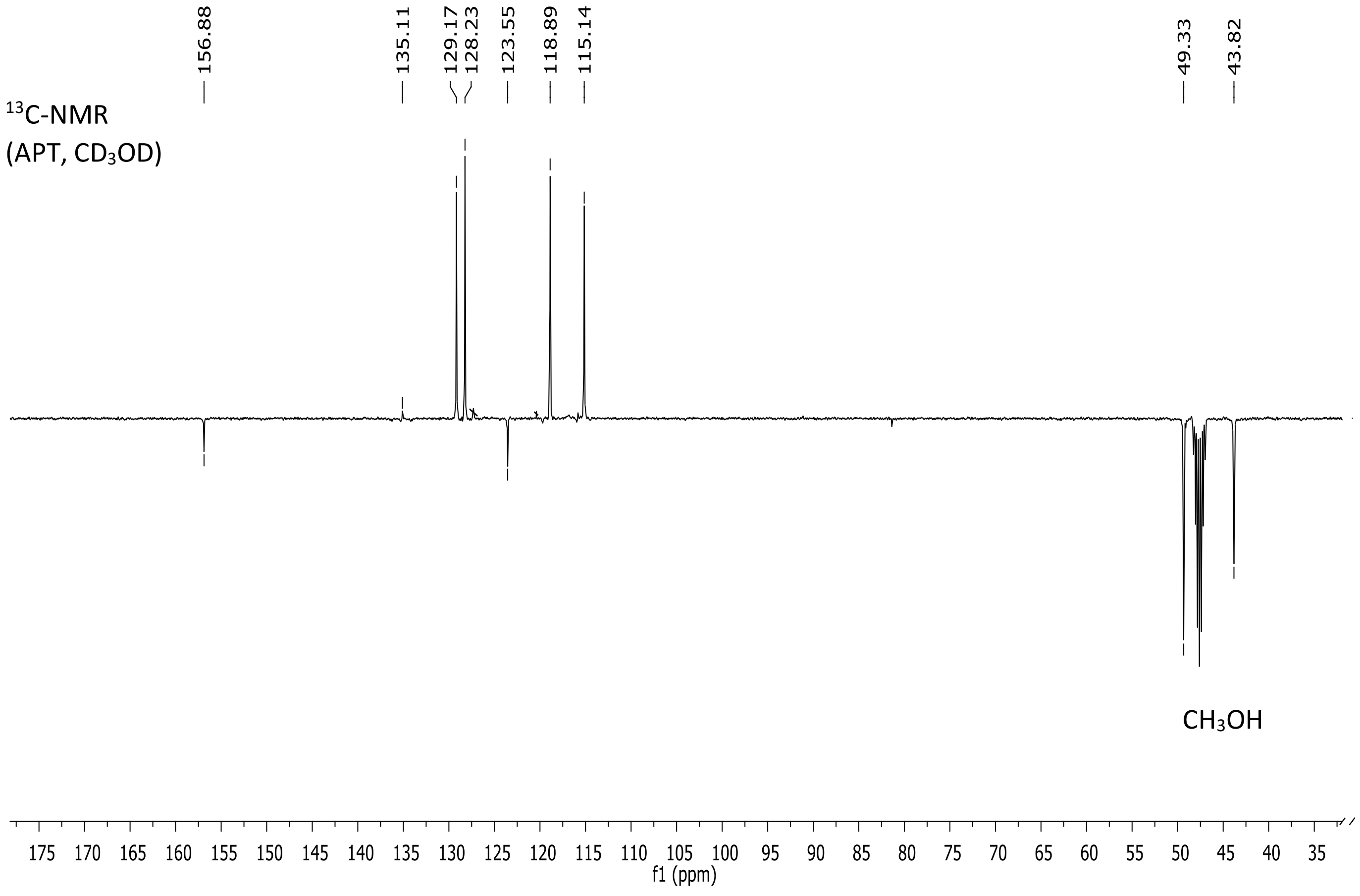
156.88
135.11
129.17
128.23
123.55
118.89
115.14
49.33
43.82
^{13}C-NMR
(APT, CD_3OD)
CH_3OH
175 170 165 160 155 150 145 140 135 130 125 120 115 110 105 100 95 90 85 80 75 70 65 60 55 50 45 40 35
f1 (ppm)

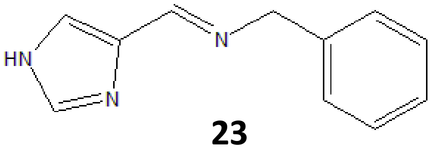

HN
N
N

23

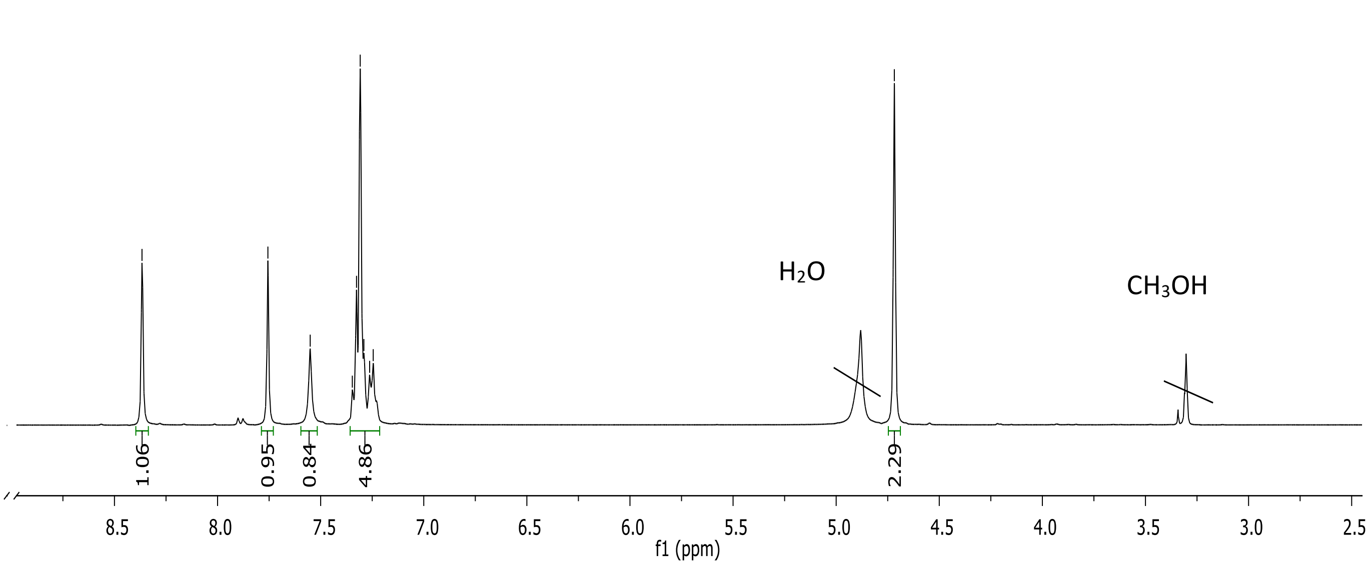

8.37
7.76
7.55
7.35
7.33
7.31
7.29
7.26
7.25
4.72
¹H-NMR (CD₃OD)
H₂O
CH₃OH
1.06
0.95
0.84
4.86
2.29
8.5 8.0 7.5 7.0 6.5 6.0 5.5 5.0 4.5 4.0 3.5 3.0 2.5
f1 (ppm)

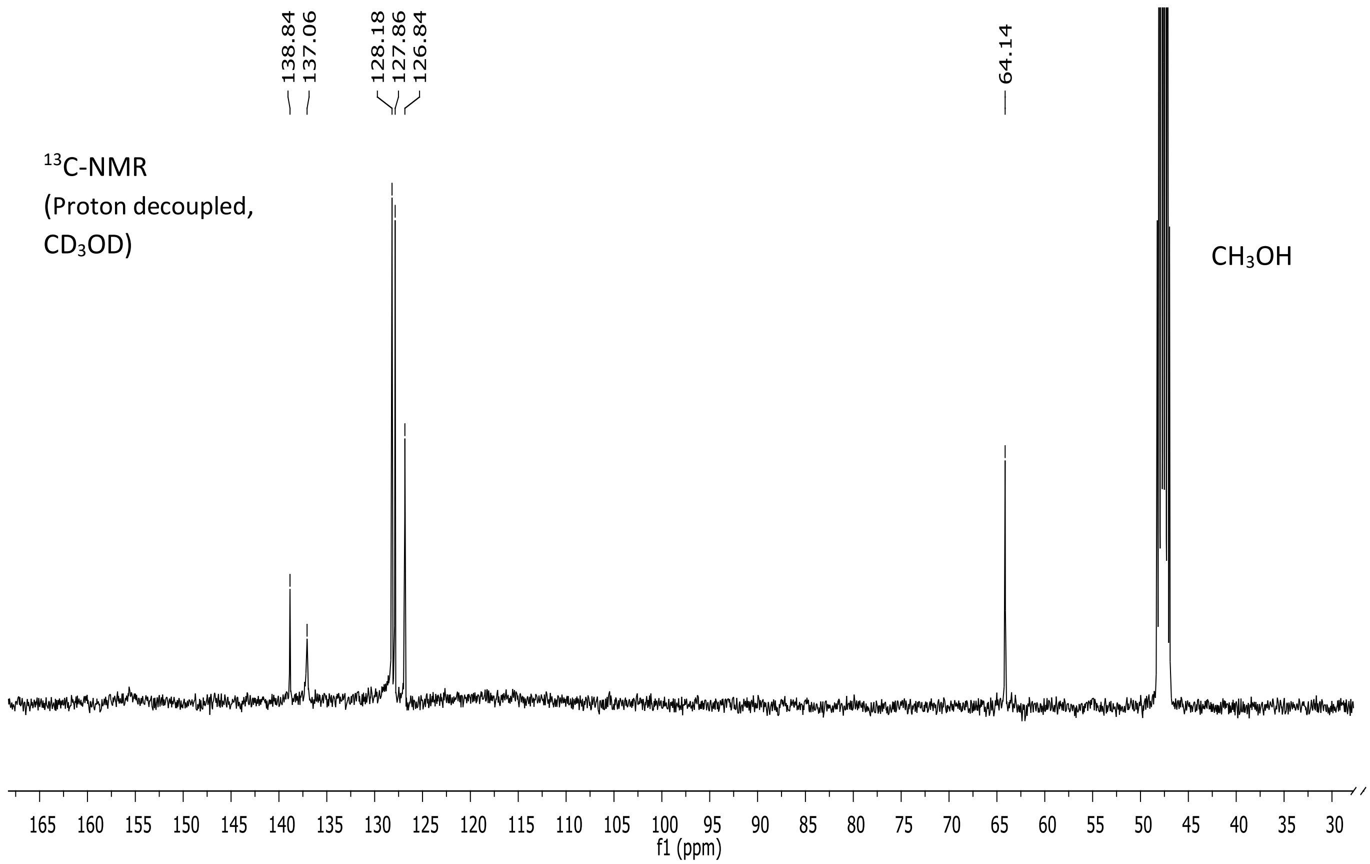

138.84
137.06
128.18
127.86
126.84
64.14
¹³C-NMR
(Proton decoupled,
CD₃OD)
CH₃OH
165 160 155 150 145 140 135 130 125 120 115 110 105 100 95 90 85 80 75 70 65 60 55 50 45 40 35 30
f1 (ppm)
